# A Neonatal Murine *Escherichia coli* Sepsis Model Demonstrates That Adjunctive Pentoxifylline Enhances the Ratio of Anti- vs. Pro-inflammatory Cytokines in Blood and Organ Tissues

**DOI:** 10.3389/fimmu.2020.577878

**Published:** 2020-09-23

**Authors:** Esther M. Speer, Elizabet Diago-Navarro, Lukasz S. Ozog, Mahnoor Raheel, Ofer Levy, Bettina C. Fries

**Affiliations:** ^1^Department of Pediatrics, Renaissance School of Medicine at Stony Brook University, Stony Brook, NY, United States; ^2^Department of Medicine, Renaissance School of Medicine at Stony Brook University, Stony Brook, NY, United States; ^3^Precision Vaccine Program, Division of Infectious Diseases, Boston Children's Hospital, Boston, MA, United States; ^4^Harvard Medical School, Boston, MA, United States; ^5^Broad Institute of MIT and Harvard, Cambridge, MA, United States; ^6^U.S. Department of Veterans Affairs, Northport VA Medical Center, Northport, NY, United States

**Keywords:** newborn, sepsis, *Escherichia coli*, anti-inflammatory, pentoxifylline, interleukin 10, cytokines

## Abstract

**Introduction:** Neonatal sepsis triggers an inflammatory response that contributes to mortality and multiple organ injury. Pentoxifylline (PTX), a phosphodiesterase inhibitor which suppresses pro-inflammatory cytokines, is a candidate adjunctive therapy for newborn sepsis. We hypothesized that administration of PTX in addition to antibiotics decreases live bacteria-induced pro-inflammatory and/or enhances anti-inflammatory cytokine production in septic neonatal mice without augmenting bacterial growth.

**Methods:** Newborn C57BL/6J mice (< 24 h old) were injected intravenously with 10^5^ colony forming units (CFUs)/g weight of a bioluminescent derivative of the encapsulated clinical isolate *Escherichia coli* O18:K1. Adequacy of intravenous injections was validated using *in vivo* bioluminescence imaging and Evans blue. Pups were treated with gentamicin (GENT), PTX, (GENT + PTX) or saline at 0, 1.5, or 4 h after sepsis initiation, and euthanized after an additional 4 h. CFUs and cytokines were measured from blood and homogenized organ tissues.

**Results:** GENT alone inhibited bacterial growth, IL-1β, and IL-6 production in blood and organs. Addition of PTX to GENT profoundly inhibited *E. coli-*induced TNF and enhanced IL-10 in blood of newborn mice at all timepoints, whereas it primarily upregulated IL-10 production in peripheral organs (lung, spleen, brain). PTX, whether alone or adjunctive to GENT, did not increase microbial colony counts in blood and organs.

**Conclusion:** Addition of PTX to antibiotics in murine neonatal *E. coli* sepsis promoted an anti-inflammatory milieu through inhibition of plasma TNF and enhancement of IL-10 production in plasma and organs without increasing bacterial growth, supporting its utility as a potential adjunctive agent for newborn sepsis.

## Introduction

Sepsis remains the leading cause of neonatal morbidity and mortality worldwide, especially among the 11% of all newborns that are born preterm (1–3). In the United States alone bacterial sepsis affects > 30,000 live births annually (4). Despite major advances in neonatal care in developed countries, 40% of septic neonates still die or suffer from major neurodevelopmental disability, and little progress has been made over the past three decades (1, 5). The distinct neonatal inflammatory immune response to severe infections, while key to reducing microbial invasion, has been associated with mortality and multiple organ injury including the brain (6–8). Adjunctive anti-inflammatory agents may mitigate the deleterious effects of the associated neonatal systemic inflammatory response syndrome, thereby improving survival and outcome (9–11). However, corticosteroids are associated with significant side effects and are generally not recommended for neonatal sepsis (12), and other adjunctive immunologic interventions for neonatal sepsis have not demonstrated clinical benefit in randomized controlled trials (10, 11). This unfortunate state of affairs is in part due to inadequate sample sizes and unique physiological, pharmacological, and ethical challenges in conducting clinical studies in this vulnerable population (11, 13). Thus, there continues to be an urgent unmet clinical need for approaches to prevent or treat hyper-inflammation associated with sepsis in the newborn.

Pentoxifylline (PTX) is a low molecular weight phosphodiesterase inhibitor that increases intracellular cyclic adenosine monophosphate (cAMP) and decreases transcription of pro-inflammatory cytokines (14). PTX is a candidate for adjunctive therapy of newborn sepsis and necrotizing enterocolitis (15, 16), which could potentially be licensed for use in neonates. Small sample sizes and risk of selection, detection and attrition biases limit the rigor of human neonatal trials thus far (15). We have demonstrated that when tested in human blood *in vitro*, PTX inhibits production of pro-inflammatory cytokines [e.g., tumor necrosis factor (TNF) and the inflammasome-mediated cytokine interleukin-1β (IL-1β)] in response to pure *Toll*-like receptor (TLR) and inflammasome agonists as well as live neonatal pathogens (17, 18). Importantly, a greater effect was observed in newborn cord blood compared to adult blood, and endogenous expression of anti-inflammatory and pro-resolution cytokines (IL-10, IL-6) (17, 19) was preserved.

Studies of the effect of PTX on preterm and term newborn animals are encouraging but thus far limited. In neonatal mice, PTX decreased death from subcutaneous *Staphylococcus aureus* infections (20). In preterm rabbits infected with aerosolized group B streptococci, PTX enhanced pulmonary clearance of bacteria and decreased inflammatory mediators in bronchoalveolar lavage fluid (21). Furthermore, PTX reduced incidence and severity of necrotizing enterocolitis in newborn rats (22). Activated leukocytes in septic patients may contribute to sustained inflammation and organ failure (23), and inflammatory cytokines and chemokines (e.g., TNF, IL-1β, CXCL-8) have been associated with perinatal brain injury (6, 24, 25). On the other hand, bone marrow stromal cells administered to aged adult mice reduced sepsis-induced mortality and improved multiple organ function via reprogramming of host monocytes and macrophages that led to increased IL-10 production (26). Treatment with either anti-TNF-α or IL-10 significantly improved survival in 1 day old neonatal mice *with E. coli* sepsis (27). Suppressing pro-inflammatory cytokines such as TNF and/or enhancing endogenous IL-10 production might therefore be beneficial in neonatal sepsis. How infection-induced systemic or local inflammatory responses can interrupt normal tissue structure and organ function such as the neonatal brain (24, 28), and how anti-inflammatory therapeutics such as PTX could restore homeostasis remains unknown. Furthermore, the effects of timing of anti-inflammatory treatment in relation to sepsis initiation (23), its interaction with antimicrobial agents (18), the relationship of systemic *vs*. organ-specific inflammation (23) and the systemic and organ-specific effects of anti-inflammatory agents such as PTX, as well as potential sex differences in response to anti-inflammatory approaches (29, 30), remain largely unknown.

Based on our *in vitro* experiments in human neonatal cord blood, wherein PTX inhibited live bacteria-induced inflammatory responses without enhancing bacterial proliferation (18), and previous reports of increased bacterial clearance with PTX (31), we hypothesized that adjunctive PTX in addition to antibiotics decreases live *E. coli*-induced pro-inflammatory and/or enhances anti-inflammatory cytokine production in blood and peripheral organs of septic neonatal mice without enhancing bacterial growth. To investigate our hypothesis and address these questions, we developed and validated a neonatal murine sepsis model, which we adapted from a previously published model of neonatal *Staphylococcus epidermidis* sepsis (32), consisting of intravenous (IV) live bacterial injections of the most frequently encountered neonatal pathogen of early-onset sepsis, namely *Escherichia coli* (33), in mice < 24 h old. We then employed this model to characterize the inhibitory efficacy of adjunctive PTX treatment in addition to antibiotics on the systemic and organ-specific inflammatory response to *E. coli* sepsis, its effects on systemic and organ-specific bacterial growth, the impact of timing of antimicrobial and/or anti-inflammatory treatment in relation to sepsis initiation on systemic and organ-specific innate immune responses, the interaction of PTX and antimicrobial treatment on these immune responses, and its potential gender-specific immunomodulatiory effects. We demonstrate for the first time that addition of PTX to gentamicin (GENT) suppresses systemic pro-inflammatory and enhances production of anti-inflammatory cytokines in blood and organ tissues without increase of bacterial burden, thus supporting the potential utility of PTX as an adjunctive anti-inflammatory agent for newborn sepsis.

## Materials and Methods

### Preparation of Microorganisms

Live *E. coli* K1 strain [# 700973, American Type Culture Collection; Manassas, VA, or a bioluminescent K1 strain A192PP-*lux2*, which was derived from the neonatal septicemia clinical isolate *E. coli* A192 by two rounds of passage through neonatal rat pups with bacterial recovery from the blood and subsequent introduction of the *lux* operon, as described by Witcomb et al. (34)] was used to induce experimental murine neonatal sepsis. Single colonies of *E. coli*, stored at 4°C on Luria-Bertani (LB) agar plates containing kanamycin (50 μg/ml), were grown overnight in LB media (Becton Dickinson; Franklin Lakes, NJ) under kanamycin pressure [since the bioluminescent derivative *E. coli* strain A192PP-*lux2* was engineered by introduction of the *lux* operon (*luxCDABE*) from the bacterium *Photorhabdus luminescens* on a disarmed mini-Tn5 transposon by conjugation under kanamycin-selection pressure (34)] in a Forma Scientific Orbital Shaker (Thermo Fisher Scientific; Waltham, MA) at 150 RPM at 37°C to stationary phase. An aliquot was then transferred to fresh growth medium at 1:100 dilution and grown (150 RPM, 37°C) to exponential phase for 2 h. After centrifugation and washing of microbial suspensions in sterile saline, bacterial colonies per ml were determined spectrophotometrically at 600 nm and confirmed by plating of serial (1:10) dilutions and manual counting of colony forming units (CFU) as described below. Microbial suspensions were diluted in sterile saline to yield the desired inoculum concentration, i.e., 4 × 10^6^ CFU per ml, thus resulting in 10^5^ CFU per g body weight when administering an injection volume of 25 μl per g weight. Gentamicin susceptibility was confirmed by plating these microorganisms onto agar plates containing different antimicrobial concentrations. The minimal inhibitory concentration was determined, which was well below the clinically relevant concentration ranges for this agent.

### Animal Model and Experimental Protocol

C57BL/6J female and male breeders were obtained from Jackson Laboratories (Bar Harbor, ME), and were mated in-house at our animal facility. Pups were delivered naturally at term gestation, and remained with their dams except for brief interruptions due to experimental procedures. Animals were fed *ad libitum* with a standard chow diet, and maintained in a year-around climate-controlled environment with a 12-h light-dark cycle. Pups of both sexes were used for all experimental procedures. BSL 2 containment was employed for all experiments involving live bacteria. The research protocol and all animal procedures were approved by the Institutional Animal Care and Use Committee at Stony Brook University, Stony Brook, NY.

#### Murine Newborn Sepsis Model

**Figure 1:**
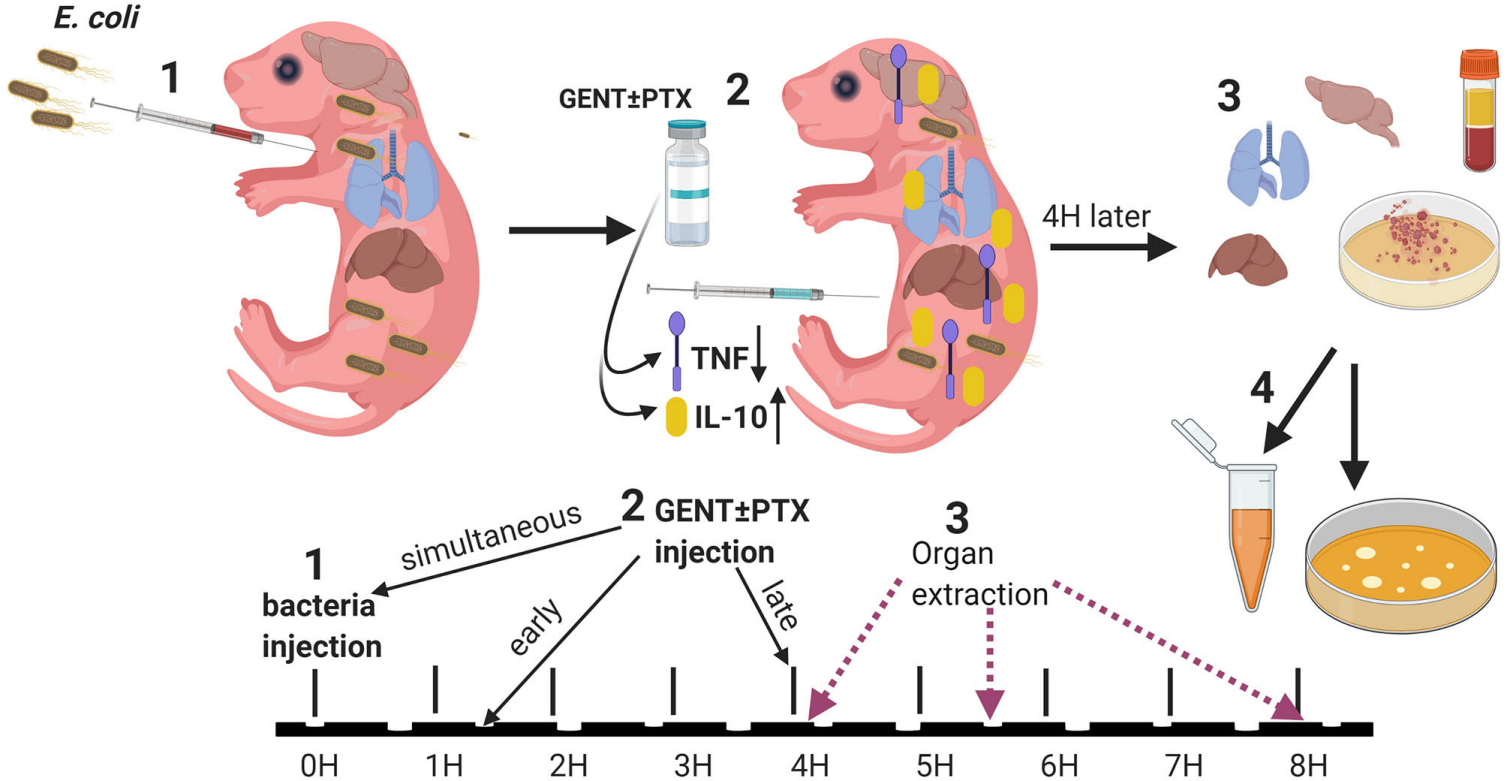
Experimental design. (1) Neonatal mice (< 24 h old) were injected with *E. coli* via the right intra-jugular route at the start of the experiment (0H time point). (2) Subsequently, pups were injected intraperitoneally with GENT, PTX, (GENT + PTX), or saline control immediately following bacteria injection (simultaneously, at the 0H time point), early (1.5H after bacteria injection), or late (4H after bacteria injection). (3) After additional 4H of incubation, i.e., at the 4H, 5.5H, or 8H time point from the start of the experiment, mice were euthanized and blood and organs obtained for (4) bacterial plating and cytokine measurements.

Newborn mice pups under 24 h old were injected IV via the external jugular route with live *E. coli* in 25 μl sterile saline per g body weight. Injections were performed with a two-person technique as previously described (32). Mice pups were manually restrained by one investigator, while the other investigator located the external jugular vein and performed the injection. In order to aid visualization and success of IV cannulation, transillumination, and magnifying glasses were used (35), in addition to brief (< 30 s) sedation/anesthesia with isoflurane open drip and Mepilex™ tape (Mölnlycke Health Care; Norcross, GA) as needed. Following the injection, mice pups were monitored for signs of distress, marked with a pen for identification, and returned to their cages once fully recovered from sedation/anesthesia. As previously reported, the intravenous injection procedure itself lasts ~1 min and is generally well-tolerated by mice pups with 100% procedure-related survival (32). For our treatment experiments, following IV bacterial injections, pups were treated with either 5 μg per g body weight gentamicin (Fresenius Kabi; Lake Zurich, IL), 60 μg per g weight PTX (VWR International, LLC; Buffalo Grove, IL), combined gentamicin and pentoxifylline, or an equal volume of sterile saline control (Hospira Inc.; Lake Forest, IL) (20 μl per g weight), administered intraperitonally (IP) once at different time points in relation to the initial bacterial inoculation. In order to investigate the effects of timing of antimicrobial and/or anti-inflammatory treatment in relation to sepsis initiation, these agents were administered either simultaneously (immediately after bacterial inoculation, 0 h delay), early (1.5 h after bacterial injections) or late (4 h after bacterial injections). All pharmacological agents used in these animal experiments were United States Pharmacopeia (USP) grade. 32 G Hamilton syringes (Hamilton Company; Reno, NV) were employed to enable dosing at 1μl precision for injections of live bacteria as well as antibiotic and anti-inflammatory agents. Following sepsis treatment, mice pups were euthanized after an additional 4 h, i.e., after 4, 5.5, or 8 h following bacterial inoculation (see Figure 1 for a schematic representation of our treatment protocol), and blood and organ tissues were obtained under sterile conditions for further processing as described below. Bacterial injections were performed in the morning, and mice were monitored for the specified durations until euthanized in the afternoon or evening.

#### Validation of Sepsis Model With Evans Blue

**Figure 2:**
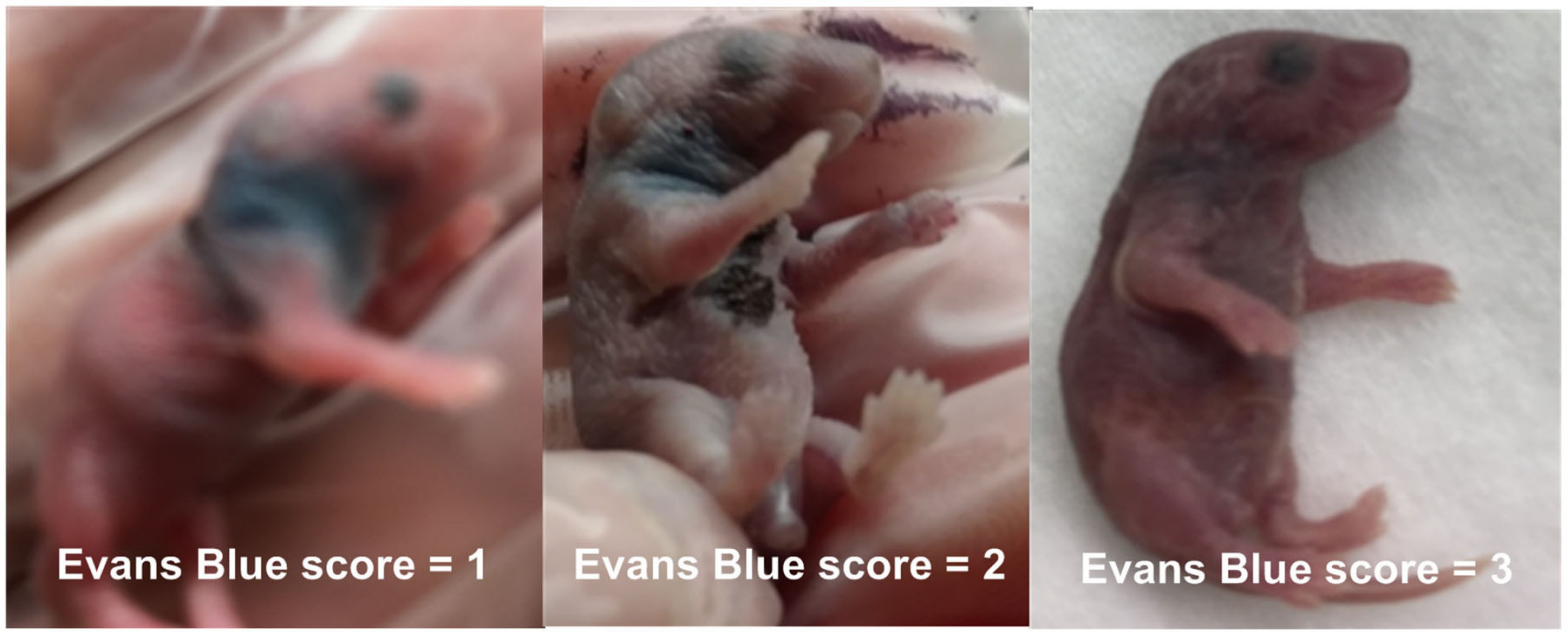
Evans blue IV injection quality score. 0.05% Evans blue dye was added to the bacterial inoculum prior to injection, and images were taken immediately after IV injection. Successful IV bacterial injections led to homogenous discoloration of neonatal mice (score = 3), partial extravasation led to local discoloration at the injection site with slight general discoloration (score = 2), and failed IV injections appeared as localized blue discoloration without general color changes (score = 1), as demonstrated on these representative photographs.

In order to confirm the successful performance of IV injections, low concentration (0.05%) Evans blue, a dye which is not detectable in the extravascular space shortly after intravenous injections, was mixed to the bacterial inoculum prior to injections (36–38). Successful IV bacterial injections led to homogenous discoloration of neonatal mice (assigned score = 3), partial extravasation led to local discoloration at the injection site with slight general discoloration (score = 2), and failed IV injections appeared as localized blue discoloration without general color changes (score = 1). Photographs of all injected animals were obtained for documentation of color changes (see representative images in Figure 2). Only Evans blue injection scores of 3 were used for further analyses for all sepsis-related treatment experiments.

#### Validation of Sepsis Model With Optical Imaging

In order to confirm the adequacy of IV bacterial injections with an objective quantitative method, in addition to the subjective semiquantitative Evans blue scoring system based on the observed color change, we utilized the bioluminescent *E. coli* K1 strain A192PP-*lux2* (34), and measured photon emission with an IVIS Lumina III *In Vivo* Imaging System (PerkinElmer; Waltham. MA) soon after IV bacterial injections. Our standard settings for optical imaging consisted of 2 min acquisition time, subject height of 0.5 cm (which provided the maximal sensitivity), and settings of 2, 4, and 8 for binning. Animals were placed supine with their right side (injection site) up and were kept under anesthesia using isoflurane 2 to 3% on the preheated instrument platform, in order to avoid any artifacts due to movements. Optical images were analyzed by placing regions of interest (ROIs) of standard size and shape over the region of the bacterial injection site of each animal image. Images were obtained for 2, 4, and 8 binning settings and the average radiance of all images was calculated for each animal. Higher concentrations of bacteria around the injection site, i.e., extravascular leakage of bacteria, led to higher photon emission around the area of the injection site on subsequent optical imaging. Using these instrument settings and based on data derived from initial images of animals with Evans blue injection scores of 3 *vs*. < 3, indicating adequate *vs*. unsuccessful IV bacterial injections, a cutoff point of equal or < 4 × 10^4^ p/s/cm^2^/sr radiance was employed in order to classify an IV bacterial injection as successful.

### Determination of Sex

The sex of neonatal mice was determined through Transnetyx Genotyping™ of tissue for the presence of the y chromosome (Transnetyx; Cordova, TN). Several undetermined samples during the initial run were subsequently repeated. For quality control purposes, 10 tissue samples from C57BL/6J mice of known male and female sex were submitted for genotyping in a blinded fashion.

### Preparation of Organ Tissues for Bacteriological Studies

After completion of predetermined incubation periods, mice were euthanized by decapitation, and blood, and organ tissues were harvested under sterile conditions. Small volume blood sampling (~20–30 μl) employed sterile capillary tubes (Drummond Scientific Company; Broomall, PA), and blood was immediately mixed with sodium citrate and kept on ice. The left lung (to minimize the possibility of bacterial contamination from potential residual extravasated microorganisms at the time of bacterial inoculation into the right external jugular vein), liver, spleen, and brain were dissected and immediately placed into 1.7 ml microcentrifuge tubes pre-filled with 500 μl sterile endotoxin-free saline and kept on ice. Upon completion of organ dissections, an aliquot of blood samples was used for bacterial plating with serial (1:10) dilutions using sterile endotoxin-free saline onto kanamycin-containing LB agar plates (Becton Dickinson). The remaining blood samples were centrifuged at 500 g for 10 min at room temperature, and plasma supernatants were carefully removed without disturbing the cell pellets and stored at −80°C. The weight of organ tissues was determined by subtracting the measured tube weights prior to the addition of organ tissues from their respective weights measured after addition of organ tissues using a Mettler Toledo AT261 Delta Range Analytical Balance (Mettler Toledo; Columbus, OH). Organ tissues were mixed with sterile 2.3 mm zirconia/silica beads (Biospec Products Inc.; Bartlesville, OK) and homogenized with a Mini-Beadbeater-16 (Biopec Products Inc.), as previously described (32). An aliquot of each tissue homogenate was then used for bacterial plating with serial (1:10) dilutions onto kanamycin-containing LB agar plates. The remaining tissue samples were centrifuged at 13,000 g for 10 min at 4 °C, and supernatants were harvested and stored at −80°C for subsequent cytokine measurements. Bacterial plates of blood and tissue homogenates were incubated at 37°C in a humidified incubator for ~18–24 h, and microbial colonies were then manually counted with an eCount Colony Counter (Heathrow Scientific; Vernon Hills, IL) as previously described (18). All colony count results were expressed as CFU counts per ml blood and CFUs per mg organ tissue, respectively.

### Measurement of Cytokine Concentrations in Plasma and Tissue Homogenates

Cytokine concentrations (TNF, IL-1β, IL-6, and IL-10) in blood plasma samples and tissue homogenate supernatants were determined with Bio-Plex Pro magnetic multiplex assays (Bio-Rad; Hercules, CA) and analyzed on the Bio-Plex 200 system with Bio-Plex Manager 5.0 software (Bio-Rad). Results were expressed as cytokine concentrations in pg per ml plasma and pg per mg protein concentration for supernatants of organ tissue homogenates. Duplicate technical replicates were used for all immunological studies. Protein concentrations in supernatant tissue homogenate samples were determined with the Bradford method (Bio-Rad Laboratories; Richmond, CA) and measured on a Spectramax 190 Plate Reader (Molecular Devices LLC; San Jose, CA).

### Statistical Analysis

All microbial counts were expressed as CFUs per ml *vs*. CFUs per mg tissue for plasma and organ tissue samples, respectively, and cytokine concentrations were expressed as pg per ml plasma *vs*. pg per mg protein for supernatant tissue homogenates. In order to account for the possibility of litter effects on these findings, cytokine concentrations of samples derived from septic animals treated with antibiotics and/or PTX were also expressed as a percentage compared to cytokine concentrations of plasma and organ tissue samples from untreated septic animals within the same litter, which were defined as 100%. Likewise, in addition to the absolute CFUs per ml or per mg tissue, CFU results from samples of septic animals that were treated with GENT, PTX, or (GENT + PTX) were also expressed as percentage change compared to those results obtained from untreated septic animals within the same litter, defined as 100%. Only samples from animals with a good Evans blue injection score of 3 were analyzed for comparisons of antimicrobial and/or anti-inflammatory treatment effects. Similarily, only samples from animals with Evans blue scores of 3 were analyzed to determine the relationships between bacterial inoculum and subsequent recovery of CFUs from blood and organ tissues as well as cytokine concentrations in these samples.

Means and standard errors were estimated for normally distributed data, and median and interquartile ranges (IQR) for non-normally distributed data, whereby the assumption of normality was assessed graphically using Q-Q-plots and through the application of Kolmogorov-Smirnov tests for normal distribution. Group comparisons between untreated and treated [GENT, PTX, or (GENT + PTX)] septic animals employed one-way ANOVA or Kruskal-Wallis tests for multiple group comparisons that were corrected by false discovery rates, for parametric and non-parametric data, respectively. Unpaired Welch *t-*tests, which do not assume variances homogeneity, and Mann Whitney *U*-tests were used for pair-wise group comparisons, as indicated. *GraphPad* Prism Version 8.4 (GraphPad Software; San Diego, CA) was used for analyses and for graphing of results. All statistical tests were two-sided and *p* < 0.05 were determined significant.

For animal ethical reasons, we used the lowest estimated number of animals for each experiment expected to achieve reliable results. Due to differences in the expected effect sizes between different groups and experimental conditions, the number of animals used varied accordingly among the different types of experiments (e.g., only few animals were required for our validation experiments that employed injections with 10-fold increasing bacterial inoculum). In order to assure adequate statistical power in light of limited numbers of experimental animals, we therefore computed a *post hoc* analysis of the achieved power to detect treatment-related group differences in bacterial CFUs and cytokine concentrations (TNF, IL-10) in blood and lung tissue between animals receiving GENT, (GENT + PTX) and saline controls (SAL), employing an alpha error of 0.05 and the given sample and effect sizes of our study. This analysis was conducted using G^*^Power software for Wilcoxon-Mann-Whitney test version 3.1 (https://www.psychologie.hhu.de/arbeitsgruppen/allgemeine-psychologie-und-arbeitspsychologie/gpower.html; Allgemeine Psychologie und Arbeitspsychologie, Heinrich Heine Universität Düsseldorf, Germany) (39).

## Results

### Validation of Murine Neonatal Sepsis Model With Evans Blue and Optical Imaging

In order to overcome the challenge of confirming successful small volume IV injections of bacteria in neonatal mice, we developed two new injection quality scoring systems based on Evans-blue dye and optical imaging of bioluminescent bacteria.

**Figure 3:**
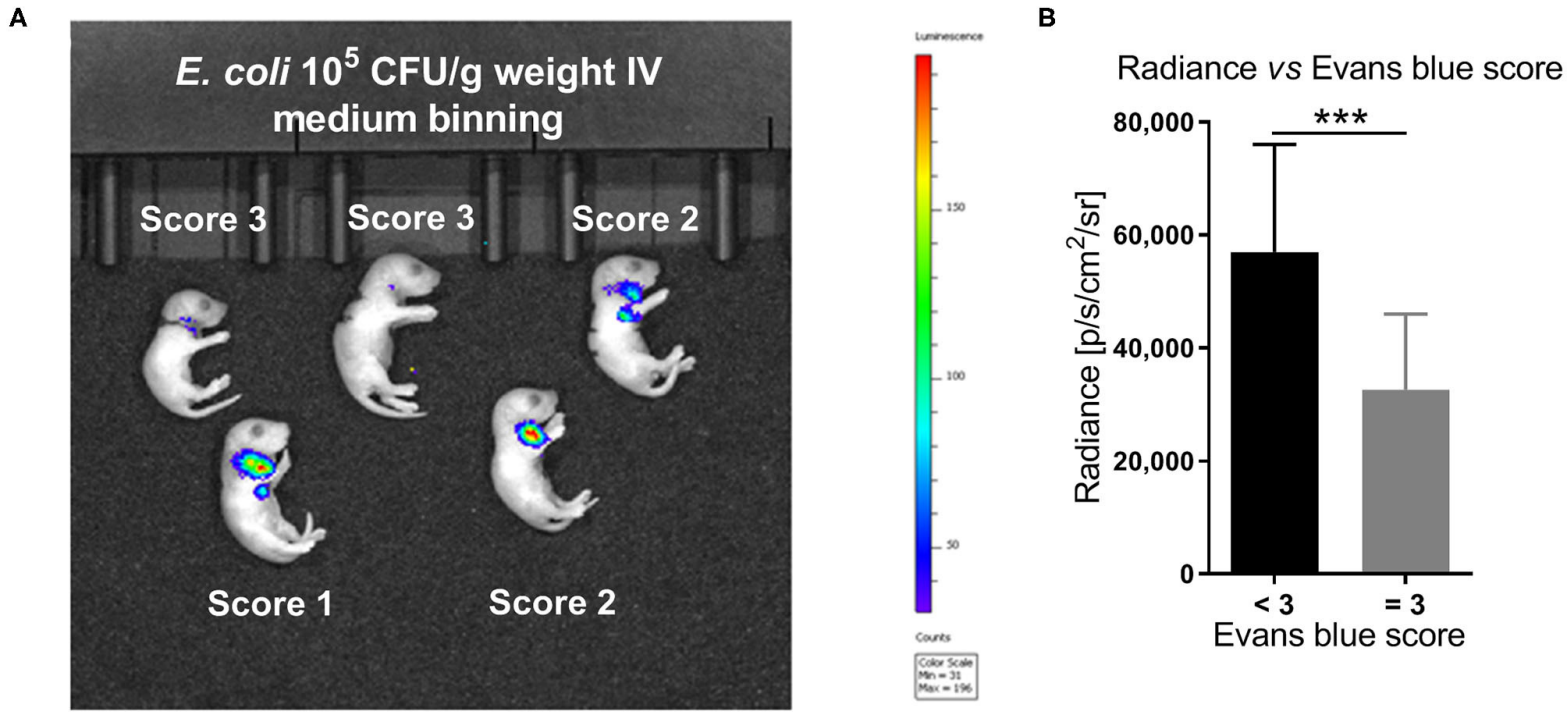
*In vivo* imaging of bioluminescent *E. coli* correlated with Evans blue IV injection score. **(A)** Optical imaging following IV injection of 10^5^ CFU per g body weight of the bioluminescent *E. coli* A192PP-*lux2* strain (34). The representative image shows the corresponding Evans blue IV injection score for each animal. **(B)** The average radiance measured shortly after IV injections correlated with the estimated Evans blue IV injection score (Spearman correlation *r* = −0.51, 95% confidence interval: −0.61 to −0.41, *p* < 0.001, *n* = 230). Significant difference between median (IQR) radiance derived from animals with Evans blue scores < 3 *vs*. =3 was as indicated: ^***^p < 0.001, 2-sided Mann-Whitney *U*-test.

As shown in the representative image and graph (Figures 3A,B), the average radiance measured shortly after IV injections correlated with the estimated Evans blue-based injection score (Spearman correlation *r* = −0.51, 95% confidence interval −0.41 to −0.61, *p* < 0.001). Furthermore, a high Evans blue injection score of 3, representing successful IV bacterial injections, resulted in significantly lower median radiance compared to Evans blue injection scores < 3 (32,567 *vs*. 56,977 p/s/cm^2^/sr, *p* < 0.001).

**Figure 4:**
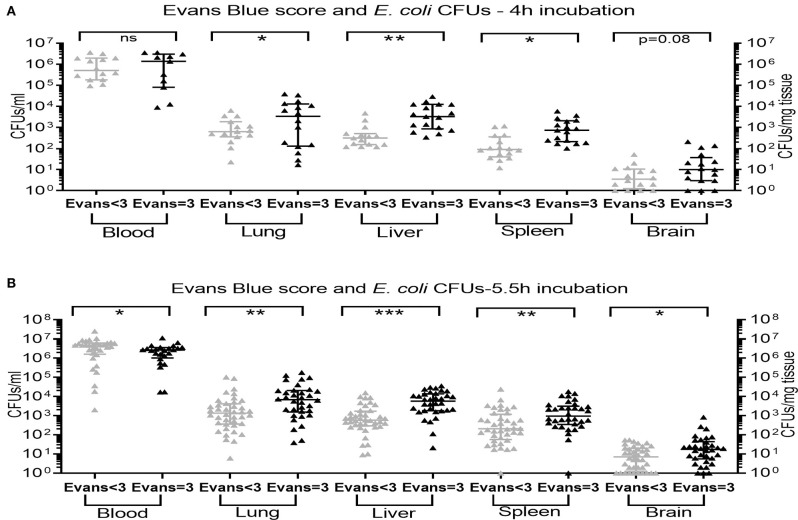
Recovery of *E. coli* CFUs in organ tissue was associated with a high Evans blue score. Neonatal mice < 24 h old were injected IV with *E. coli* 10^5^ CFU/g body weight suspended in saline with 0.05% Evans blue dye. Evans blue injection scores were assigned immediately following IV injections. After **(A)** 4 h (*n* = 11–17) and **(B)** 5.5 h (*n* = 24–40) of incubation, mice were euthanized and blood and homogenized organ tissue serially plated for bacterial counting. Significant differences of CFU counts for each organ and blood (plotted as median and IQR) derived from animals with low Evans blue score < 3 *vs*. high Evans blue score = 3 (successful IV injection) were as indicated: ^*^p < 0.05, ^**^p < 0.01, ^***^p < 0.001, 2-sided *t-*tests with Welch's correction **(A)** and Mann-Whitney *U*-tests **(B)**.

An Evans blue injection score of 3, representing successful IV injection, was associated with significantly higher bacterial CFUs in all peripheral organs tested at 5.5 h after sepsis initiation (Figure 4B), and in the lung, liver, and spleen at 4 h after injection of bacteria with a trend toward higher CFU counts in brain tissue for Evans blue injection scores of 3 as compared to injection scores < 3 (*p* = 0.08) (Figure 4A). Comparable CFU counts in blood samples observed after good Evans blue injection scores *vs*. scores below 3 at 4 h after sepsis initiation (Figure 4A) are likely due to dissemination of bacteria from the blood to peripheral organs. This interpretation is further suggested by the finding of significantly decreased CFU counts in blood after good Evans blue scores compared to low scores at 5.5 h after bacterial injections, whereas CFUs in all peripheral organs were increased in animals with high *vs*. low injection scores at that timepoint (Figure 4B). CFU counts in lung and liver tissue achieved after bacterial injections with a high Evans blue score of 3 remained significantly elevated after 8 h of incubation as compared to CFU counts in these organ tissues after bacterial injections with low Evans blue scores (*p* < 0.05) (*data not shown*). Of note, the addition of Evans blue did not alter the viability of the bacteria used in this study nor the bacteria-induced cytokine production in human blood tested *in vitro* (*data not shown*).

#### Effect of Bacterial Inoculum on CFU Counts and Cytokine Production in Blood and Organ Tissue in Newborn Mice

**Figure 5:**
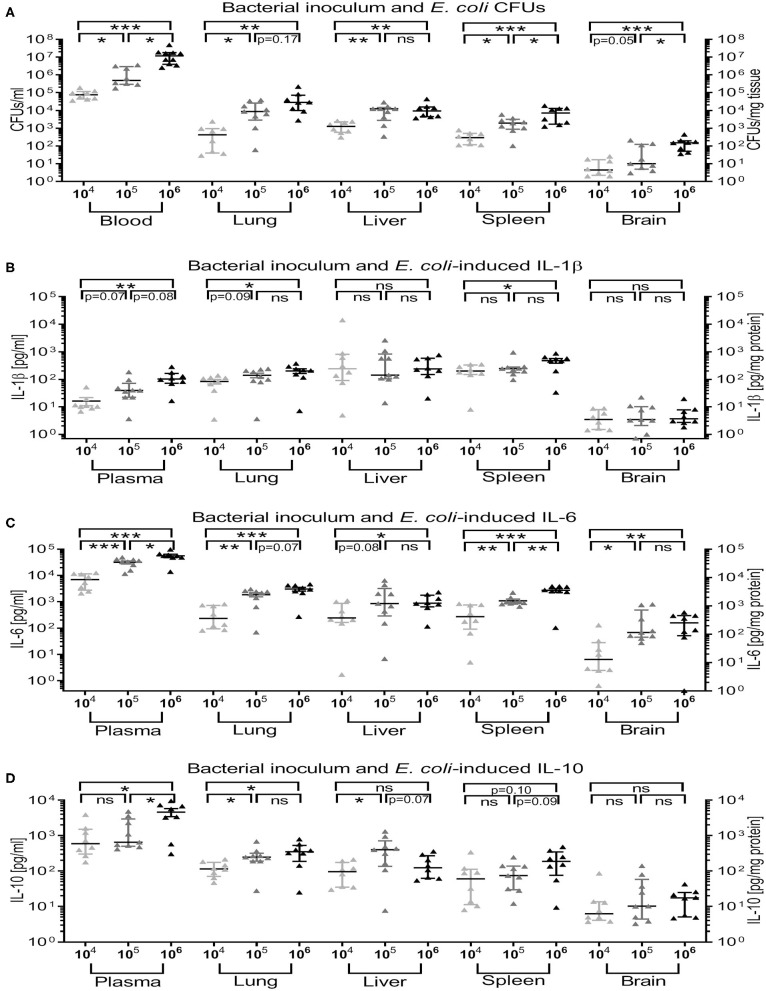
Recovery of *E. coli* CFUs and magnitude of cytokine production in blood and organ tissue was inoculum-dependent. Neonatal mice < 24 h old were injected IV with *E. coli* 10^4^, 10^5^, or 10^6^ CFU/g body weight suspended in saline with 0.05% Evans blue. After 4 h of incubation, mice were euthanized and blood, and homogenized organ tissue serially plated for bacterial counting, and cytokine concentrations measured. Mean (± SEM) or median (IQR) were plotted and one-way ANOVA employed. Significant differences of **(A)** CFU counts (*n* = 5–7 each), **(B)** IL-1β, **(C)** IL-6, and **(D)** IL-10 cytokine concentrations (*n* = 8–9 each) for each organ and blood or plasma from animals injected with different bacterial loads were as indicated: ^*^p < 0.05, ^**^p < 0.01, ^***^p < 0.001, ns, non-significant; Kruskal-Wallis tests **(A)** or one-way ANOVA **(B–D)** corrected for false discovery rate.

Among animals with an Evans blue score of 3, i.e., animals with successful IV bacterial injection, *E. coli* CFUs from newborn mice blood and organ tissue cultures were all bacterial inoculum-dependent (10^4^, 10^5^, and 10^6^ CFUs per g weight; Figure 5A) CFU counts. Furthermore, these were significantly associated with inoculum-dependent production of IL-1β in blood, lung, and spleen (Figure 5B), IL-6 in blood and all organ tissues tested (Figure 5C), and IL-10 in blood, lung, and liver with a non-significant trend in spleen tissue (Figure 5D). These data confirm the validity and feasibility of our model, i.e., successful injection of increasing bacterial inocula leads to increased recovery of bacterial CFUs accompanied by enhanced inflammatory responses in blood and peripheral organs of experimentally infected newborn mice.

#### Standardization of Optical Imaging of Bioluminescent *E. coli* Injections in Neonatal Mice

In order to determine the cut-off value for good radiance representing successful IV bacterial injection, neonatal mice were injected subcutaneously into their right neck area with decreasing loads of bioluminescent *E. coli*. The average radiance measured shortly after injections correlated highly with the injected bacterial inoculum (Spearman correlation *r* = +0.91, *p* < 0.001) (see Supplementary Figure 1A). Furthermore, the median radiance of mice injected with 3.3 ×10^3^ CFUs/g weight subcutaneously differed significantly from the measured median radiance after injection of 10^5^ CFUs/g weight (median: 4.7 × 10^5^ p/s/cm^2^/sr, IQR: 2.6 × 10^5^ to 6.7 × 10^5^ p/s/cm^2^/sr) as well as 3.3 × 10^4^ CFUs/g weight, which correspond to 100% and 33% of the bacterial load used for IV injections in our sepsis mouse model. The measured median radiance after injection of 10^4^ CFU/g weight (median 3.1 × 10^4^ p/s/cm^2^/sr, IQR: 2.3 × 10^4^ to 3.5 × 10^4^ p/s/cm^2^/sr), which corresponds to 10% of the total injected bacterial inoculum for IV injections in this study, still showed a non-significant trend toward lower values compared to 10^5^ CFUs/g weight. We thus determined a radiance of 4 × 10^4^ p/s/cm^2^/sr as cut-off value (depicted as interrupted line in Supplementary Figure 1A), which corresponds to ~10% of the bacterial inoculum used, below which an IV injection was determined acceptable for inclusion in our study. Since the measured radiance also depends on the ROI chosen, we employed ROIs of standard size and shape, which were placed over the injection site on the corresponding optical images as shown in the example in Supplementary Figure 1B.

On the basis of the above findings, we thus defined successful IV injections in neonatal mice as Evans blue score of 3 or as measured radiance ≤ 4 × 10^4^ p/s/cm^2^/sr, and only animals fulfilling these criteria were included in the analyses of all subsequent sepsis-related treatment experiments. These therapeutic experiments all employed 10^5^ CFUs/g weight of bioluminescent *E. coli* delivered in a volume of 25 μl/g weight, i.e., 4 × 10^6^ CFUs/ml, and optical imaging was performed upon completion of IV injections.

### Characteristics of Mice in Treatment Experiments of Neonatal Sepsis

A total of 227 pups (39.5% male, 60.5% female) with a mean (± SEM) weight of 1.5 ± 0.02 g were used to investigate treatment effects of GENT and/or PTX in *E. coli* sepsis on their first day of life. Since our study examined the short-term effects of adjunctive anti-inflammatory treatment of neonatal murine sepsis on bacterial colony counts and cytokine production in blood and organs, the overall mortality among experimental animals was low. All simultaneously treated pups survived the observation period. Among early treated neonatal mice, only one out of 27 (3.7%) pups [male, treated with (GENT + PTX)] died, whereas a total of 9 (12%) late treated pups did not survive the intended duration of observation. These consisted of 2 (10%, 2 males) saline-controls, 2 (10.5%, 2 females) GENT-treated, 2 (10.5%, 1 male and 1 female) (GENT + PTX)-treated, and 3 (17.6%, 3 females) PTX alone-treated septic mice. Due to these low overall mortality numbers, further analyses and group comparisons could not be performed. However, this indicated that addition of PTX to GENT did not increase mortality in neonatal murine *E. coli* sepsis compared to GENT alone, a finding that requires further investigation under experimental conditions specifically designed to study the mortality risk of adjunctive PTX use and longer observation periods. Although it remains to be determined if the 17.6% mortality among septic mice treated with PTX alone might be relevant, prudent use of PTX under appropriate antibiotic coverage in cases of potential neonatal sepsis would be warranted.

### Microbial Colony Counts in Blood and Organ Tissue of Newborn Mice With Experimental *E. coli* Sepsis

**Figure 6:**
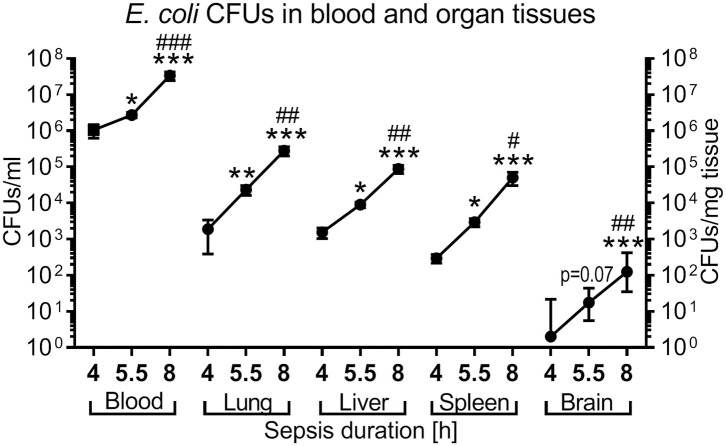
Time-dependent *E. coli* bacterial growth pattern in murine neonatal sepsis. Neonatal mice < 24 h old were injected IV with *E. coli* 10^5^ CFU/g body weight. After 4 h (*n* = 10), 5.5 h (*n* = 34), and 8 h (*n* = 19) of incubation, mice were euthanized and blood, and homogenized organ tissue serially plated for bacterial counting. Mean (± SEM) or median (IQR) CFUs were plotted for each time point as indicated. Significant differences of CFU counts for each organ and blood recovered after increasing incubation periods were as follows: ^*^p < 0.05, ^**^p < 0.01, ^***^p < 0.001 for 4 *vs*. 5.5 h and 4 *vs*. 8 h comparisons; ^#^*p* < 0.05, ^*##*^*p* < 0.01, ^*###*^*p* < 0.001 for 5.5 *vs*. 8 h comparisons, Kruskal-Wallis tests corrected for false discovery rate.

Next we tested if PTX increases the replication of microorganisms, which would prohibit its clinical use. We quantified growth of *E. coli* in blood and peripheral organ tissue of newborn mice intravenously infected with 10^5^ CFUs per g body weight, followed by immediate (0 h), early (1.5 h), or late (4 h) treatment with GENT, PTX, (GENT + PTX) or saline control (SAL) administered IP. As shown in Figure 6, *E. coli* CFUs recovered from blood and all organ tissues (lung, liver, spleen, and brain) of untreated (SAL) septic neonatal mice significantly increased over time (4, 5.5, and 8 h). CFU counts in blood samples were very high already at 4 h after sepsis initiation (mean of 1.1 × 10^6^ CFU per ml), and further increased to more than 30-fold after 8 h of sepsis duration. CFU counts of untreated neonatal mice after 4 h of sepsis were moderately high in lung and liver (mean of 1.9 × 10^3^ and 1.5 × 10^3^ CFU per mg tissue, respectively), and low in spleen and brain tissue (mean of 297 and 11 CFU per mg tissue, respectively). However, tissue CFU counts increased rapidly with increasing sepsis duration, showing an ~50-fold increase of mean CFUs in liver tissue, ~150-fold higher mean CFUs in lung, ~170-fold higher mean CFUs in spleen, and ~60-fold increased median CFUs per mg tissue in the brain (Figure 6).

**Figure 7:**
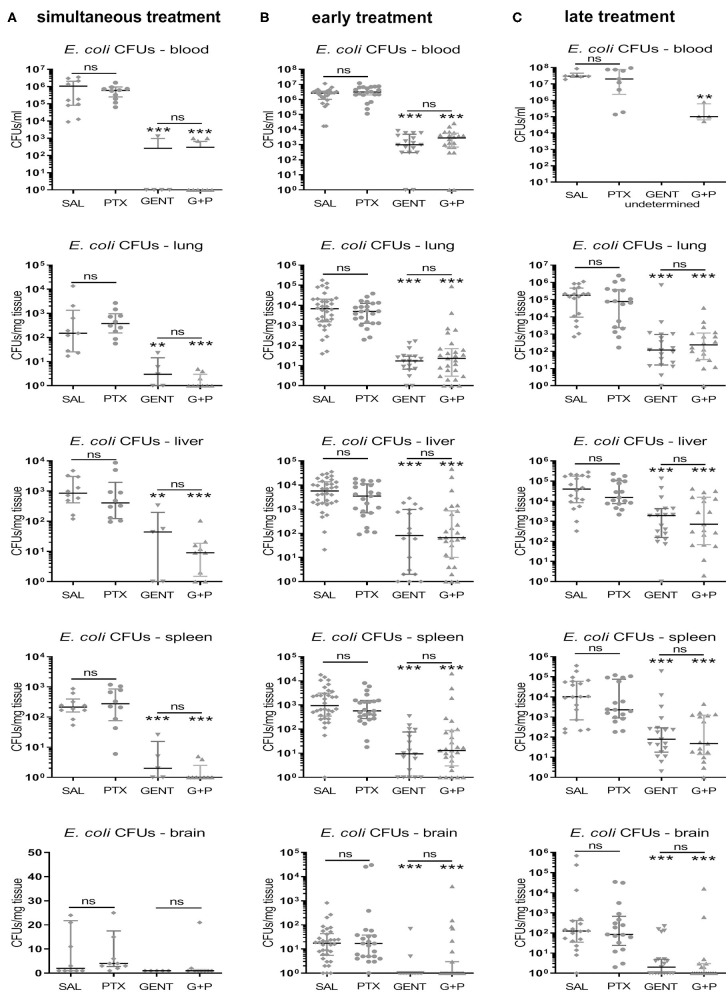
PTX did not increase *E. coli* bacterial growth in neonatal mice. Neonatal mice < 24 h old were injected IV with *E. coli* 10^5^ CFU/g body weight, followed by **(A)** simultaneous (0 h, *n* = 9–10), **(B)** early (1.5 h, *n* = 17–34), or **(C)** late (4 h, *n* = 17–19) IP injection of saline control (SAL), GENT, PTX, or GENT + PTX (G + P). After an additional 4 h of incubation, i.e., 4, 5.5, or 8 h from the time of sepsis initiation, mice were euthanized and blood and homogenized organ tissue serially plated for bacterial counting. Median (IQR) CFU counts for each organ and blood recovered after each treatment condition and time point are shown. Significant differences between SAL *vs*. each treatment condition as well as between GENT *vs*. (G + P) were as indicated: ^**^p < 0.01, ^***^p < 0.001, ns, non-significant; 2-sided Mann-Whitney *U*-tests. No results available for late treatment with GENT for blood due to insufficient samples.

As expected, GENT significantly diminished CFU counts in blood and all tested organ tissues, whether administered simultaneously, early, or late in relation to bacterial inoculation (Figure 7). Addition of PTX to GENT in neonatal mice infected with *E. coli* did not change their CFU counts derived from blood and peripheral organ tissues (lung, liver, spleen, and brain) at all three time points investigated. Likewise, simultaneous, early, and late PTX treatment alone without antimicrobial agents did not increase CFUs in blood and organ tissues compared to untreated septic neonatal mice after 4, 5.5, and 8 h of sepsis (Figures 7A–C), suggesting that PTX does not affect microbial proliferation and/or viability *in vivo*. *Post hoc* analysis for an alpha error of 0.05 revealed a power > 99% to detect differences between (GENT + PTX)-treated *vs*. control animals for CFUs in blood and 87% for CFUs in lung tissue. Based on our sample sizes, a difference in effect sizes between GENT- *vs*. (GENT + PTX)-treated animals of 0.77 for CFUs in blood samples and 0.71 for CFUs in lung tissue would have been detectable with a power of 80% and an alpha error of 0.05.

### Pro- and Anti-inflammatory Cytokine Production Show Characteristic Time-Dependent Systemic and Organ-Specific Patterns During Murine Neonatal Sepsis

**Figure 8:**
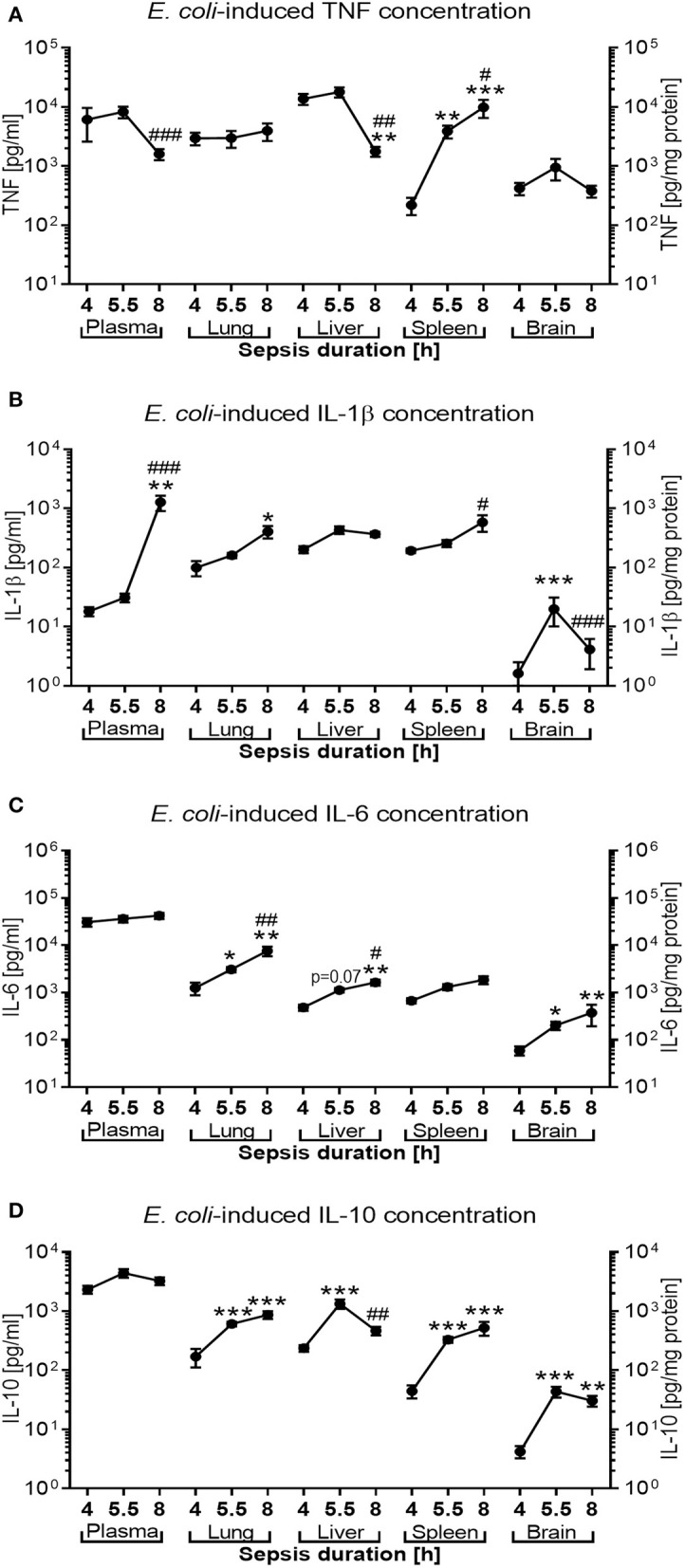
*E. coli*-induced cytokine production in neonatal mice as a function of sepsis duration. Neonatal mice < 24 h old were injected IV with *E. coli* 10^5^ CFU/g body weight. After 4 h (*n* = 8), 5.5 h (*n* = 34), and 8 h (*n* = 18) of incubation, mice were euthanized, and pro- and anti-inflammatory cytokines were measured in plasma and homogenized organ tissue. Mean (± SEM) or median (IQR) concentrations for **(A)** TNF, **(B)** IL-1β, **(C)** IL-6, and **(D)** IL-10 were plotted for each time point as indicated. Significant differences were as follows: ^*^p < 0.05, ^**^p < 0.01, ^***^p < 0.001 for 4 *vs*. 5.5 h and 4 *vs*. 8 h comparisons; ^#^*p* < 0.05, ^*##*^*p* < 0.01, ^*###*^*p* < 0.001 for 5.5 *vs*. 8 h comparisons, Kruskal-Wallis tests corrected for false discovery rate.

*E. coli* induced high concentrations of both pro- and anti-inflammatory cytokines in untreated septic newborn mice (Figure 8). Whereas, plasma concentrations for TNF (mean concentration of 6,142 pg/ml), IL-6 (31,000 pg/ml), and IL-10 (2,322 pg/ml) were already very high after 4 h of sepsis duration and subsequently remained elevated, IL-1β plasma concentrations increased only after 5.5 h of sepsis initiation to levels comparable to the other cytokines (mean concentrations of 1,273 pg/ml after 8 h). By contrast, plasma TNF concentrations as an early responding pro-inflammatory cytokine started to trend down after 8 h of sepsis duration (mean 1,607 pg/ml, *p* < 0.001) (Figure 8A). Comparable to the time course of TNF in plasma, TNF concentrations in lung and liver tissue were already high at 4 h of sepsis duration and significantly decreased in liver tissue by 8 h after sepsis initiation. TNF in spleen tissue, on the other hand, showed a different time course for production of this pro-inflammatory cytokine, which was characterized by initially relatively low levels of TNF (mean 220 pg/mg protein) that then rapidly increased to 3,901 pg/mg protein by 5.5 h and 9,908 pg/mg protein after 8 h of sepsis duration. Brain tissue reached its peak tissue TNF concentration (mean 950 pg/mg protein) after 5.5 h of sepsis duration.

IL-1β concentrations in lung and spleen tissue mirror the delayed increase of this cytokine in plasma, whereas cerebral tissue levels of this cytokine remain low with a peak mean concentration of 33 pg/mg protein after 5.5 h of sepsis duration (Figure 8B). While IL-6 plasma concentrations remained steadily elevated, IL-6 tissue concentrations in peripheral organs (lung. liver, and brain) continued to rise between 4 and 8 h after sepsis initiation (Figure 8C). Similarily, whereas IL-10 plasma concentrations remained at a high level throughout the observation period, peripheral tissue concentrations of this anti-inflammatory cytokine increased in all organs studied after 4 h of sepsis duration and began to decline in liver tissue after 8 h of sepsis (mean of 1,335 *vs*. 467 pg/mg protein, *p* < 0.01, after 5.5 and 8 h of sepsis duration, respectively) (Figure 8D).

### Addition of PTX to Antimicrobial Treatment Inhibits *E. coli*-Induced TNF Production in Blood and Enhances Anti-inflammatory IL-10 in Blood and Peripheral Organs of Septic Newborn Mice

**Figure 9:**
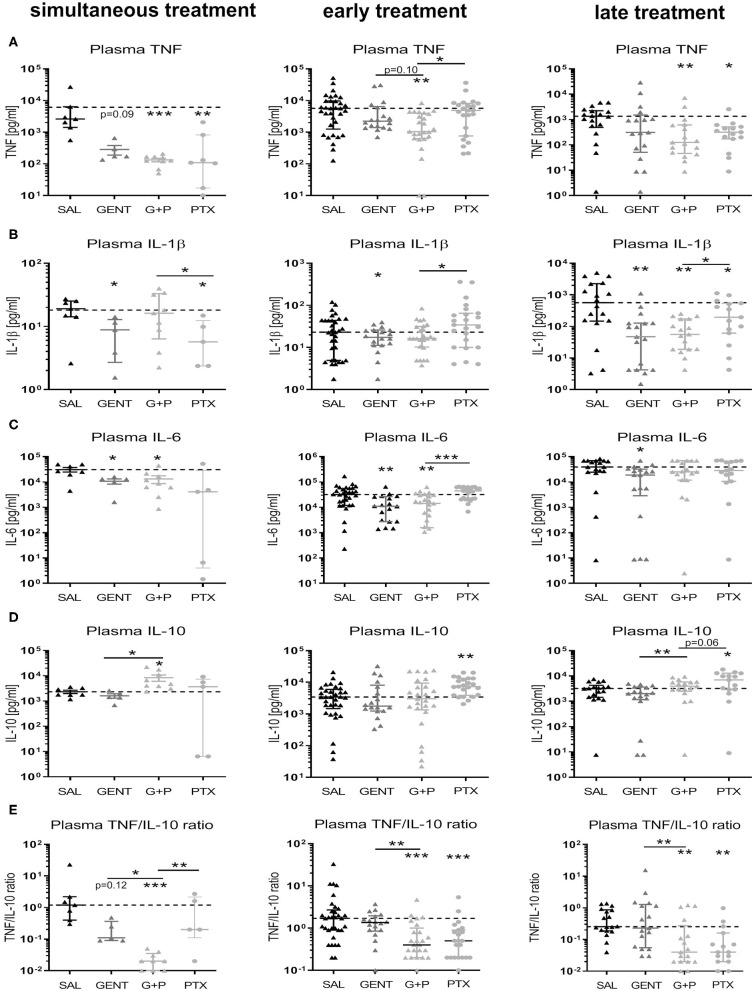
Addition of PTX to GENT profoundly inhibited *E. coli*-induced plasma TNF and enhanced plasma IL-10 in septic newborn mice. Neonatal mice < 24 h old were injected IV with *E. coli* 10^5^ CFU/g body weight, followed by simultaneous (0 h, *n* = 7–9), early (1.5 h, *n* = 18–33), or late (4 h, *n* = 15–18) IP injection of SAL, GENT, PTX, or GENT + PTX (G + P). After an additional 4 h of incubation, i.e., 4, 5.5, or 8 h from the time of sepsis initiation, mice were euthanized and plasma cytokines were measured. Mean (± SEM) or median (IQR) plasma cytokine concentrations for **(A)** TNF, **(B)** IL-1β, **(C)** IL-6, **(D)** IL-10, and **(E)** TNF-to-IL-10 ratios after each treatment condition and time point are shown. Significant differences between SAL *vs*. each treatment condition as well as between GENT *vs*. (G + P) and PTX *vs*. (G + P) were as indicated: ^*^p < 0.05, ^**^p < 0.01, ^***^p < 0.001, one-way ANOVA with Welch's correction or Kruskal-Wallis tests corrected for false discovery rate.

Addition of adjunctive PTX to GENT in septic newborn mice significantly and profoundly inhibited TNF plasma concentrations compared to untreated saline control animals with simultaneous and early treatment, with a non-significant trent for decreased concentrations compared to GENT alone [mean TNF plasma concentrations for SAL, GENT, and (GENT + PTX) of 6,142 *vs*. 285 *vs*. 133 pg/ml with simultaneous, and 8,180 *vs*. 5,868 *vs*. 2,128 pg/ml with early treatment], and still decreased TNF plasma concentrations compared to saline controls after late treatment (Figure 9A). By contrast, GENT alone only led to a non-significant decrease of plasma TNF after simultaneous treatment. GENT alone, on the other hand, significantly diminished IL-1β concentrations in plasma of septic mice with simultaneous, early and late treatment, without any additional inhibitory effect of adjunctive PTX (Figure 9B). Likewise, IL-6 production in plasma of septic newborn mice was significantly diminished with simultaneous, early and late GENT, whereas addition of PTX did not show any further inhibitory effects (Figure 9C). Addition of PTX to GENT enhanced the production of the anti-inflammatory IL-10 in septic neonatal mice compared to GENT alone (~5-fold increase in mean IL-10 plasma concentrations) as well as untreated saline controls (~3.6-fold increase) after simultaneous treatment, and compared to GENT alone (~2-fold increase) after late treatment (Figure 9D). Similarily, early and late treatment with PTX alone significantly enhanced plasma IL-10 production compared to untreated saline controls, with a non-significant trent toward higher plasma IL-10 concentrations compared to (GENT + PTX)-treated septic neonatal mice after late treatment, indicative of a strongly enhancing effect of PTX on IL-10 production that might partially be counteracted in the presence of GENT. Consistent with the plasma TNF-inhibiting and IL-10-enhancing actions of adjunctive PTX in addition to GENT in our murine neonatal sepsis model, (GENT + PTX) decreased the plasma TNF-to-IL-10 concentration ratio compared to GENT alone as well as untreated saline controls with simultaneous, early and late treatment in relation to sepsis initiation (Figure 9E), whereas GENT alone only resulted in a non-significant decline in the plasma TNF-to-IL-10 ratio after simultaneous treatment that immediately followed the injection of bacteria. According to these findings, the addition of PTX to GENT in septic newborn mice thus shifts their plasma cytokine production profile toward an anti-inflammatory milieu that might mitigate the bacteria-induced inflammatory response syndrome associated with *E. coli* sepsis.

**Figure 10:**
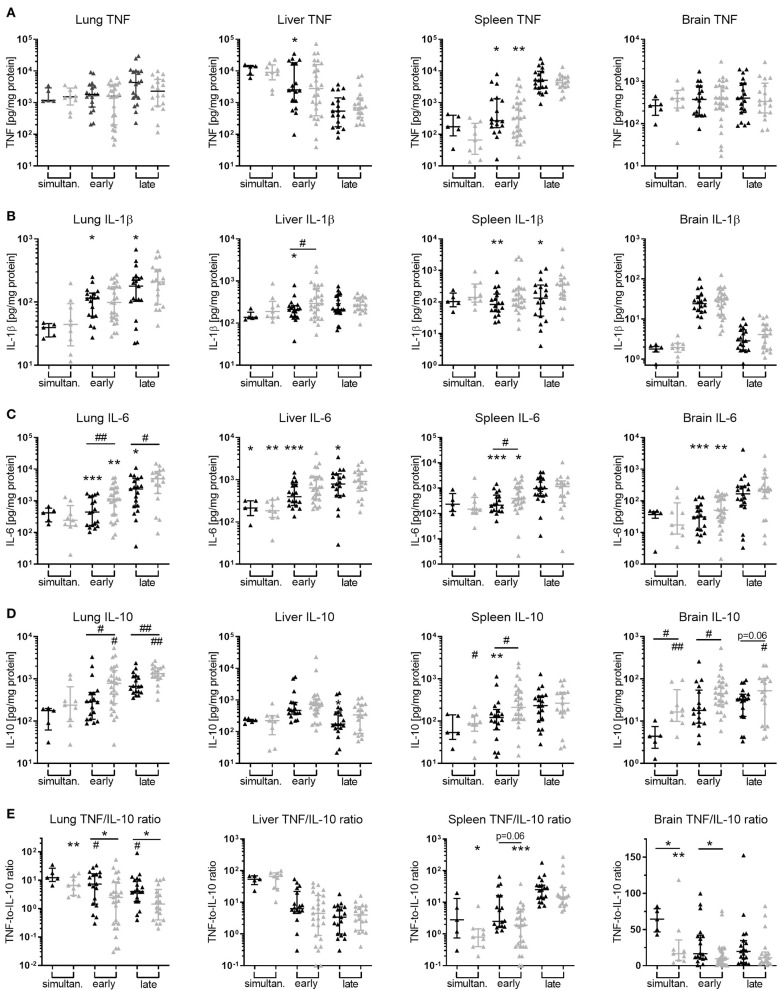
Addition of PTX to GENT enhanced *E. coli*-induced IL-10 production in peripheral organ tissues. Neonatal mice < 24 h old were injected IV with *E. coli* 10^5^ CFU/g body weight, followed by simultaneous (0 h, *n* = 7–9), early (1.5 h, *n* = 18–34), or late (4 h, *n* = 19–20) IP injection of SAL, GENT, or (GENT + PTX). After an additional 4 h of incubation, i.e., 4, 5.5, or 8 h from the time of sepsis initiation, mice were euthanized and homogenized organ tissue **(A)** TNF, **(B)** IL-1β, **(C)** IL-6, **(D)** IL-10, and **(E)** TNF-to-IL-10 for lung, liver, spleen, and brain were measured. Mean (± SEM) or median (IQR) cytokine concentrations for GENT (black bars) and (GENT + PTX) (gray bars) for each time point are shown. Significant differences of each treatment condition *vs*. untreated controls and between GENT *vs*. (GENT + PTX) were as indicated: significant increase ^*^p < 0.05, ^**^p < 0.01, ^***^p < 0.001; significant decrease ^#^*p* < 0.05, ^*##*^*p* < 0.01, 2-sided *t*-tests with Welch's correction **(A–D)** or Mann-Whitney *U*-tests **(E)** as indicated.

While adjunctive PTX to GENT exerted inhibitory actions on *E. coli*-induced plasma TNF production in neonatal mice (achieved power of 95%), addition of PTX to antibiotics did not alter TNF production in peripheral organ tissues such as lung, liver, spleen, and brain of our experimental animals (Figure 10A). Similar to its effect on IL-1β and IL-6 in plasma of septic newborn mice, GENT alone decreased production of these two cytokines in lung, liver, and spleen tissue, and inhibited IL-6 but not IL-1β production in brain tissue compared to saline control animals (Figures 10B,C). Of note, cerebral IL-1β concentrations were low compared to other organ tissues. Adjunctive PTX in addition to GENT, on the other hand, increased IL-10 tissue concentrations compared to saline control and GENT alone in lung (achieved power of 82%), spleen, and brain tissue (Figure 10D). However, compared to GENT alone, (GENT + PTX) did not modify hepatic IL-10 production nor the TNF-to-IL-10 production ratio in liver tissue of septic neonatal mice. Although GENT effectively reduced *E. coli* CFUs in lung tissue (Figure 7), GENT alone led to an increased TNF-to-IL-10 production ratio in the lung of septic neonatal mice, i.e., a shift toward a more pro-inflammatory state, compared to untreated septic mice. Addition of PTX to GENT after 1.5 and 4 h of sepsis duration, on the other hand, reduced the TNF-to-IL-10 ratio in lung tissue and effectively prevented this pro-inflammatory response observed with GENT treatment alone at the later stages of sepsis in our model (Figure 10E). Likewise, adjunctive PTX to GENT when administered simultaneous with sepsis initiation significantly decreased the TNF-to-IL-10 concentration ratio in the lung compared to saline controls. (GENT + PTX) significantly reduced the ratio of TNF-to-IL-10 concentrations in spleen tissue as compared to untreated septic animals, and in cerebral tissue as compared to GENT alone as well as untreated septic newborn mice (Figure 10E). Since TNF concentrations in lung and brain tissue of infected mice were not modified by the addition of PTX to antibiotics, the observed shift toward an anti-inflammatory milieu was primarily achieved through upregulation of IL-10 production in these organs of our *E. coli*-septic newborn mice. Adjunctive PTX in addition to GENT for *E. coli* sepsis appears to exert its anti-inflammatory effects in plasma through inhibition of TNF and enhancement of IL-10 production, whereas it primarily exerts its actions through upregulation of IL-10 production in peripheral organ tissues including the lung and brain.

### Relative Treatment-Related Changes of Bacterial CFUs and Cytokine Concentrations Compared to Untreated Septic Neonatal Animals

**Figure 11:**
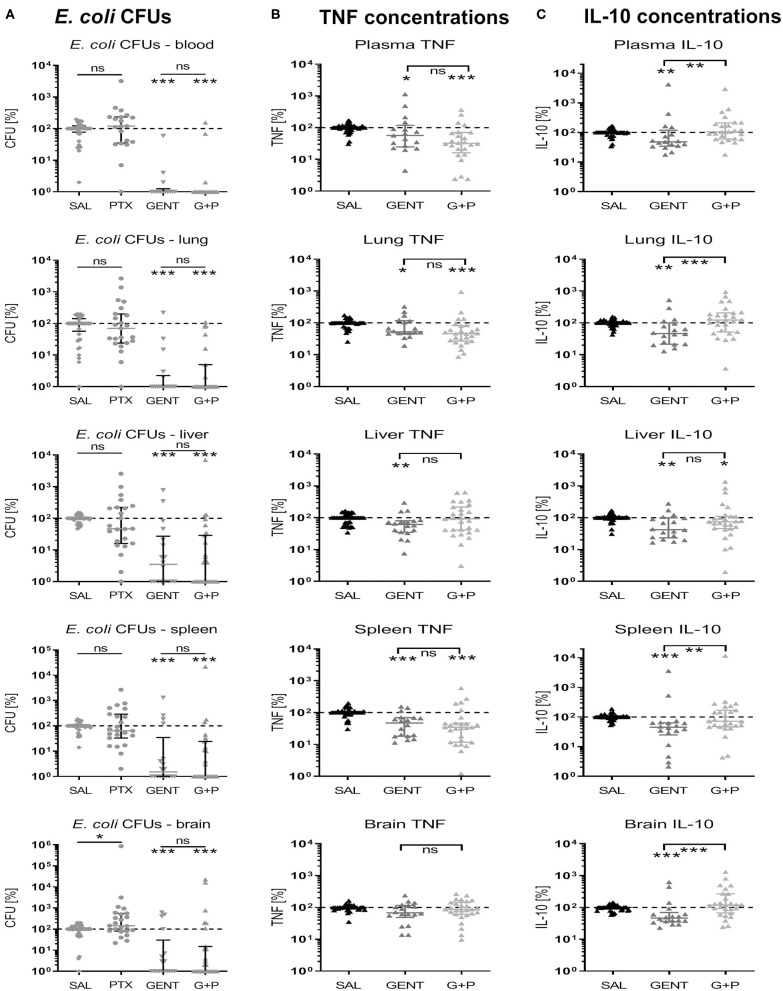
Effect of GENT, PTX, and (GENT + PTX) on *E. coli* CFUs and cytokine production in neonatal mice relative to untreated septic mice. Neonatal mice < 24 h old were injected IV with *E. coli* 10^5^ CFU/g body weight, followed by 1.5 h delayed IP injection of SAL, GENT, PTX, or GENT + PTX (G + P). After an additional 4 h of incubation mice were euthanized, blood and homogenized organ tissues serially plated for bacterial counting, and cytokine concentrations measured. **(A)** CFU counts, **(B)** TNF, and **(C)** IL-10 concentrations for each organ and blood or plasma from animals subjected to different treatment conditions were expressed as a percentage compared to untreated septic mice within the same litter (defined as 100%). Significant differences of relative median (IQR) CFUs and cytokine concentrations of animals treated with antibiotics and/or anti-inflammatory agents *vs*. untreated controls and between GENT- *vs*. (G + P)-treated animals were as follows: ^*^p < 0.05, ^**^p < 0.01, ^***^p < 0.001, ns, non-significant; Mann-Whitney *U* **(A)** and Kruskal-Wallis tests with correction for false discovery rate **(B,C)**, *n* = 15–27.

As shown in Figure 11A, *E. coli* CFU counts for GENT- and (GENT + PTX)-treated as compared to untreated septic neonatal mice at 5.5 h after sepsis initiation were comparable between absolute CFUs and relative percentage changes of colony counts, except for increased relative CFU counts in brain tissue among PTX-alone treated compared to untreated mice. Absolute and relative TNF concentrations between GENT- and (GENT + PTX)-treated *vs*. untreated mice were also comparable, except for decreased relative but not absolute TNF concentrations in lung tissue after GENT or (GENT + PTX)-treatment and in plasma after GENT alone (Figure 11B). Likewise, absolute and relative IL-10 concentrations between GENT- and (GENT + PTX)-treated *vs*. untreated septic animals remained comparable, except for decreased relative IL-10 in plasma, lung, liver, and brain of GENT-alone treated mice, elevated relative plasma IL-10 in (GENT + PTX)-treated *vs*. GENT alone treated pups, and decreased relative IL-10 in the liver of (GENT + PTX)-treated septic neonatal mice compared to saline controls (Figure 11C). These findings demonstrate that absolute cytokine data therefore is more conservative compared to relative treatment-related effects on cytokine production in plasma and organ tissues. Since the TNF-to-IL-10 production ratios were derived from the absolute cytokine values, litter effects are unlikely to be relevant. These data support therefore the conclusion that (GENT + PTX) treatment of *E. coli*-infected neonatal mice significantly diminished the TNF-to-IL-10 concentration ratios in plasma and all organ tissues except the liver as compared to GENT alone and/or saline controls, thus indicating a shift toward an anti-inflammatory milieu through addition of PTX to antimicrobial therapy in murine neonatal sepsis.

### *E. coli* CFUs and Cytokine Production in Murine Neonatal Sepsis Analyzed by Sex

To determine if sex affects immune responses to immunomodulatory therapies, we analyzed bacterial growth patterns as well as *E. coli*-induced cytokine responses in blood and organs of male and female septic neonatal mice that were untreated or treated with antimicrobials and/or anti-inflammatory agents. Due to low numbers sex-specific analysis had to be limited to the early treatment groups only. *E. coli* CFU counts showed no significant differences between male and female mice among any of the treatment groups (see Supplementary Table 1). Likewise, TNF and IL-10 concentrations were similar among male and female untreated and treated [GENT or (GENT + PTX)] septic mice. The pro-inflammatory cytokine IL-1β, however, demonstrated significantly higher (1.6-fold) tissue concentrations in the lung of untreated, 2.5-fold increased concentrations in plasma of GENT-treated, and 2.2-fold higher concentrations in the spleen of GENT-treated female mice, and higher concentrations in the lung (1.7-fold) and spleen (1.7-fold) of (GENT + PTX)-treated male mice after 5.5 h of sepsis (Table 1). Additionally, GENT-treated female mice had 4-fold higher IL-6 in lung, whereas (GENT + PTX)-treated male mice presented with higher IL-6 concentrations in lung (~2.5-fold), spleen (2.3-fold), and brain tissues (6-fold) at 5.5 h after sepsis initiation compared to their female counterparts (Table 1). Mice treated with PTX alone revealed no significant differences in cytokine concentrations in any of the organs when analyzed by sex (see Supplementary Table 2).

**Table 1:** Effects of early GENT and PTX on *E. coli*-induced cytokines in murine neonatal sepsis by sex.

**Cytokine**	**Organ**	**Sex**	**SAL control**				**GENT**				**GENT** **+** **PTX**			
			***N***	**Mean**	**± SEM**	**Median**	***N***	**Mean**	**± SEM**	**Median**	***N***	**Mean**	**± SEM**	**Median**
TNF	Plasma	Fem.	21	7,214	± 1,701	5,845	13	7,289	± 2,828	2,439	13	2,072	± 646	1,046
		Male	12	9,870	± 4,239	4,810	5	1,914	± 347	1,619	12	2,189	± 528	1,451
	Lung	Fem.	22	2,795	± 1,093	777	13	2,668	± 808	1,536	14	2,202	± 495	1,669
		Male	12	1,942	± 959	568	5	2,188	± 655	1,860	13	1,751	± 464	1,497
	Liver	Fem.	22	19,338	± 4,425	11,697	13	10,214	± 3,205	3,420	14	12,658	± 5,265	4,362
		Male	12	17,057	± 5,652	6,003	5	5,198	± 4,041	1,226	13	7,362	± 3,075	2,780
	Spleen	Fem.	22	3,331	± 730	2,099	13	1,426	± 642	316	14	752	± 317	158
		Male	11	5,284	± 2,492	2,281	5	884	± 769	122	13	1,037	± 437	341
	Brain	Fem.	22	973	± 556	335	13	516	± 135	357	14	392	± 86	733
		Male	12	848	± 236	657	5	465	± 139	413	13	734	± 217	308
IL-1β	Plasma	Fem.	21	33	± 6	25	13	23^**^	**±** **3**	23^**^	13	22	± 6	16
		Male	12	27	± 10	15	5	9^**^	**±** **3**	11^**^	12	23	± 4	18
	Lung	Fem.	22	187^*^	**±** **22**	168^*^	13	120	± 18	120	14	87^*^	**±** **16**	62^*^
		Male	12	114^*^	**±** **18**	118^*^	5	85	± 11	84	13	153^*^	**±** **21**	172^*^
	Liver	Fem.	22	479	± 89	336	13	261	± 51	216	14	481	± 153	247
		Male	12	350	± 65	360	5	186	± 40	207	13	486	± 118	354
	Spleen	Fem.	22	285	± 49	190	13	197	± 64	118^**^	14	452	± 230	80^*^
		Male	11	200	± 32	196	5	42	± 8	53^**^	13	397	± 159	254^*^
	Brain	Fem.	22	35	± 16	18	13	32	± 8	22	14	30	± 5	27
		Male	12	30	± 8	20	5	26	± 3	26	13	36	± 9	33
IL-6	Plasma	Fem.	21	38,724	± 7,874	35,492	13	19,065	± 4,950	12,267	13	12,044	± 3,581	4,832
		Male	12	31,756	± 8,472	26,226	5	6,452	± 2,505	3,130	12	22,542	± 5,596	17,685
	Lung	Fem.	22	3,178	± 490	2,583	13	874^*^	**±** **177**	870	14	966^*^	**±** **202**	1,026^*^
		Male	12	2,879	± 746	2,755	5	220^*^	**±** **51**	173	13	2,384^*^	**±** **508**	2,465^*^
	Liver	Fem.	22	1,225	± 166	882	13	630	± 112	495	14	914	± 291	520
		Male	12	950	± 167	697	5	304	± 55	247	13	951	± 188	822
	Spleen	Fem.	22	1,381	± 245	1,177	13	458	± 120	335	14	465^*^	**±** **149**	301^*^
		Male	11	1,182	± 287	890	5	170	± 38	187	13	1,085^*^	**±** **251**	985^*^
	Brain	Fem.	22	166	± 44	120	13	57	± 12	49	14	50^*^	**±** **13**	31^*^
		Male	12	269	± 86	148	5	15	± 4	13	13	304^*^	**±** **18**	111^*^
IL-10	Plasma	Fem.	21	4,042	727	3,445	13	5,439	1,741	2,270	13	7,477	± 2,563	2,190
		Male	12	5,066	± 1,662	3,344	5	7,475	± 6,273	1,244	12	5,550	± 1,815	4,159
	Lung	Fem.	22	566	± 56	485	13	489	± 151	302	14	1,165	± 405	464
		Male	12	689	± 124	588	5	777	± 633	164	13	1,078	± 264	913
	Liver	Fem.	22	996	± 118	1,045	13	952	± 335	515	14	2,274	± 1,589	797
		Male	12	1,957	± 627	1,326	5	1,312	± 1,013	385	13	1,279	± 633	699
	Spleen	Fem.	22	352	± 53	233	13	204	± 80	138	14	444	± 196	131
		Male	11	286	± 50	261	5	205	± 133	98	13	435	± 96	318
	Brain	Fem.	22	40	± 13	25	13	33	± 9	19	14	92	38	32
		Male	12	51	± 12	45	5	60	± 50	9	13	58	± 10	47

*Neonatal mice < 24 h old were injected IV with E. coli 10^5^ CFU/g body weight, followed by early (1.5 h) IP injection of SAL, GENT, or (GENT + PTX). After an additional 4 h of incubation, i.e., 5.5 h from the time of sepsis initiation, mice were euthanized, and plasma, and homogenized organ tissue cytokines were measured. Mean (± SEM) and median cytokine concentrations in pg/ml (plasma) or pg/mg protein for each organ and treatment condition and separate for both sexes were calculated. Significant sex differences (in bold) employing 2-sided t-tests and Mann-Whitney U-tests were indicated as follows*:

* *p < 0.05*,

** *p < 0.01*.

## Discussion

The immune system of preterm and term neonates has traditionally been viewed as immature and more susceptible to infections and adverse outcomes resulting from damage caused by invading microorganisms. However, recent findings support the concept of the immune-mediated damage framework (40), specifically that the infection-induced neonatal host response contributes to organ damage including brain injury (6, 24, 28, 41, 42), suggesting that appropriately formulated and timed anti-inflammatory agents such as the phosphodiestarease inhibitor PTX may be of benefit to reduce harmful hyper-inflammation in the newborn. To investigate the actions of PTX or alternative anti-inflammatory agents in newborns, who express distinct innate immunity and suffer the greatest burden of infection (3), we further developed an intravenous live bacterial neonatal mouse sepsis model starting from the first post-natal day of life, followed by anti-microbial and anti-inflammatory treatment (32), and rigorously validated this model by applying two new IV quality scoring systems. Whereas the Evans blue dye-based method to evaluate the quality of IV injections in neonatal rodents is easily applicable to any IV injection including non-microbial injections without the need for any additional equipment, it remains subjective and dependent on user training, and therefore potentially prone to bias. Review of images of experimental animals injected with Evans blue by a trained investigator, or comparison of Evans blue scores with optical imaging results, as performed in this study, can mitigate the risk of bias. Optical imaging to verify the quality of IV bacterial injections in a quantitative and objective manner, on the other hand, besides the need for a suitable bioluminescent microbial strain requires determining a cut-off radiance value for successful IV injections. This cut-off value depends in part on the bioluminescent strain itself, as well as the volume and concentration of the injected bacterial inoculum, the site of injection, the size and position of the animal during optical imaging, as well as the instrument settings and the selected measurement region of interest. Once such a cut-off point has been determined and standard experimental settings are established, optical imaging provides a user-independent and quantitative comparable method to determine the quality of IV injections in neonatal rodents. Depending on the experimental requirements, both quality scoring systems may therefore be employed to verify quality IV injections in rodent pups in future studies.

Applying IV injection quality scoring systems to a murine neonatal sepsis model, our findings support the hypothesis that PTX decreased live *E. coli*-induced systemic inflammatory cytokine production and enhanced the production of the anti-inflammatory IL-10 in blood of newborn mice without increasing bacterial proliferation. This *in vivo* finding is consistent with our previous report on the TNF- suppressive and IL-10-enhancing effects of PTX added to GENT in human cord blood exposed to live *E. coli in vitro* (18). Likewise, these results on reduction of pro-inflammatory cytokine production especially TNF in blood and organs are consistent with other adult and neonatal animal studies. For instance, PTX injection prior to intravenous injection of live *E. coli* in adult rats decreased plasma TNF, IL-1β, and IL-6 concentrations (43). PTX-treated preterm rabbits infected with aerosolized group B streptococci had lower levels of lysozyme and TNF in bronchoalveolar lavage fluid compared to untreated controls (21). Combined PTX and indomethacin treatment of neonatal piglets with group B streptococcal sepsis led to significant reduction of TNF serum levels (44). Other studies have demonstrated that PTX also reduced bacterial endotoxin-induced TNF, interferon γ, and other acute phase reactants in young sheep (45), reversed cerebral ischemia-reperfusion-induced TNF and IL-6 levels in adult rats (46), and protected against LPS-induced white matter injury in the developing rat brain (47), suggesting that PTX can inhibit microbial-induced as well as sterile inflammation.

To our knowledge ours is the first study that demonstrated a PTX-induced enhancement of IL-10 production in a neonatal sepsis model *in vivo*, suggesting that this anti-inflammatory mechanism may be relevant in peripheral organ tissues especially during the later phases of sepsis. This novel finding also mirrors our results with newborn cord blood stimulated with purified TLR agonists and live microbes. There PTX inhibited lipopolysaccharide-induced *TNF* mRNA but enhanced *IL10* mRNA expression in newborn cord blood (17), and addition of PTX to antibiotics inhibited *E. coli*-induced pro-inflammatory cytokine production of TNF and IL-1β without decreasing IL-10 (18). Similarly, PTX inhibited TLR agonist-induced pro-inflammatory cytokines in newborn and adult blood (48), consistent with its enhancement of the cAMP-dependent pathway (14). Both anti-TNF-α and recombinant murine IL-10 injected subcutaneously improved survival of *E. coli* sepsis in neonatal mice (27), indicating a role of IL-10 in improved outcome of sepsis in these animals. In contrast to the other organs studied, addition of PTX to GENT did not inhibit hepatic TNF, and did not modify IL-10 production nor the TNF-to-IL-10 ratio in liver tissue of septic neonatal mice compared to GENT alone, an observation that warrants further investigation. PTX might protect against lipopolysaccharide-induced liver injury (49), however this has not been demonstrated for bacterial sepsis-induced hepatic inflammation.

*The kinetics of E. coli*-induced cytokine concentrations demonstrated striking differences between plasma and peripheral organ tissue responses to murine neonatal sepsis. Whereas TNF appeared to rise early in plasma as well as several peripheral organs (lung and liver), there was a time delay in the pro-inflammatory response of other organs such as the spleen and the brain. Absolute cytokine concentrations in plasma as well as changes in cytokine concentrations are therefore not necessarily predictive of pro-inflammatory cytokines in peripheral organs, which might exhibit a delayed inflammatory response. Thus, characteristic plasma and organ-specific pro- and anti-inflammatory cytokine concentrations and time-course of elevated cytokine levels are encountered during neonatal murine sepsis that need to be accounted for when investigating immunomodulatory treatment strategies that aim to reduce the sepsis-induced inflammatory response syndrome. The fact that adjunctive PTX to GENT inhibited TNF and increased IL-10 concentrations in plasma, but primarily promoted IL-10 production in peripheral organ tissues emphasizes such differences between the systemic and organ responses to immunopharmacological interventions, and might be a consequence of differences in the time-course and stage of sepsis between systemic and peripheral compartments. The effects of treatment on the sepsis-induced inflammatory response in blood and organ tissues were each determined 4 h after PTX and GENT treatment, i.e., at the 4 h time point for simultaneous treatment, at 5.5 h for early, and at 8 h for late treatment (see Figure 1), thus providing an equal period of time for treatment effects, and enabling the study of treatment effects during different phases of the inflammatory response to sepsis. *In vitro* studies of microbe-stimulated blood samples as a model of experimental sepsis are useful as a screening tool for the identification of potential candidate adjunctive anti-inflammatory agents (18, 50), but may not be entirely predictive of inflammatory immune responses in peripheral organs, as activated leukocytes in septic patients may contribute to sustained inflammation in peripheral organs und subsequent organ damage (23).

Of particular interest are the responses to sepsis in the murine neonatal brain. The changes in cerebral tissue CFU counts from very low to absent after 4 h of sepsis to moderately high CFU loads at later time-points suggest that the blood-brain barrier initially protected the brain from invading microorganisms, but lost its protective effects with progressing sepsis, thus allowing cerebral microbial invasion. Despite this delay in cerebral microbial invasion by *E. coli* in neonatal septic mice, TNF concentrations were already elevated after 4 h of sepsis and did not significantly change with further progression of sepsis, indicating that systemic pro-inflammatory cytokines such as TNF can enter cerebral tissue in the early stages of sepsis while the blood-brain barrier might still provide protection against invading microorganisms. Cerebral IL-10, on the other hand, remained initially low, thus resulting in the highest TNF-to-IL-10 concentration ratios in the brain compared to the other organ tissues investigated in this study. Addition of PTX to GENT in neonatal sepsis, which resulted in significantly diminished TNF-to-IL-10 ratios in cerebral tissue of *E. coli*-infected mice in our study, might therefore be of particular benefit to protect the vulnerable neonatal brain from sepsis-induced inflammatory injury.

While GENT alone decreased IL-1β and IL-6 concentrations in plasma and all organs tested except for cerebral IL-1β, it exerted only limited decreases in TNF concentrations in the spleen and liver with early treatment, and did not augment IL-10 concentrations nor diminish the ratio of TNF-to-IL-10 in any of the compartments. Adjunctive PTX to GENT, on the other hand, did not demonstrate any additional inhibitory effect on IL-1β and IL-6 in plasma and organ tissues, but actually increased IL-6 concentrations in lung and spleen tissue of *E. coli* septic neonatal mice, which is consistent with its previously reported enhancing effect of this pro-resolution cytokine, bearing in mind that IL-6, for example, reduces tissue neutrophilia (17, 19, 51). By contrast, adjunctive PTX to GENT significantly inhibited TNF plasma concentrations at all treatment time points tested and spleen TNF with early treatment, and enhanced IL-10 in plasma, lung, spleen, and brain tissues with resulting reduction in TNF-to-IL-10 concentration ratios demonstrating a shift toward an anti-inflammatory milieu. GENT alone even diminished IL-10 in the spleen and elevated the TNF-to-IL-10 ratio in lung tissue, thereby promoting pro-inflammatory responses, which were effectively prevented by the addition of PTX to GENT. Since GENT and PTX both exert different immunomodulatory effects, with GENT primarily decreasing IL-1β and PTX inhibiting TNF and enhancing IL-10, it is conceivable that the combination effect of PTX and an appropriate antimicrobial agent such as GENT provides the greatest suppression of sepsis-induced pro-inflammatory cytokine responses, as demonstrated in our murine neonatal sepsis model. This observation has potential clinical implications, i.e., it is possible that PTX in the context of infections including sepsis may be most helpful when used as an adjunctive agent in combination with appropriate antimicrobial therapy. The observation that concurrent administration of subtherapeutic doses of amphotericin B and a PTX analog led to increased survival times in experimental candidiasis in mice (52) further supports the potential benefit of combined antimicrobial and anti-inflammatory sepsis therapy. On the other hand, TNF may be beneficial for host immunity at low bacterial inoculum and harmful at higher inoculum, and TNF blockade with monoclonal antibodies may be beneficial or harmful depending on the microbial inoculum and strain involved (53–56). The effects of TNF-suppressing anti-inflammatory agents such as PTX in addition to antibiotics in sepsis should therefore be investigated with different bacterial loads and strains prior to its recommendation as adjunctive sepsis therapeutic.

We did not observe any increase of bacterial growth with PTX in our murine neonatal *E. coli* sepsis model, which is consistent with our previous *in vitro* studies (18) as well as published *in vivo* reports, wherein PTX even enhanced clearance of GBS from infected preterm rabbits (21) and improved bacterial clearance in the context of hemorrhage and endotoxemia in rabbits (31). While PTX decreased mortality from experimental *S. aureus* infection in newborn mice at lower doses, high dose PTX actually increased mortality in *S. aureus*-infected newborn mice (20), and led to greater fungal burden and shortened survival in murine *Candida albicans* sepsis (57). Based on limited clinical trials in human septic neonates, PTX may decrease mortality in newborn sepsis when administered in addition to antibiotics (15). Adjunctive PTX, when added to appropriate antibiotics and at recommended doses, therefore does not appear to compromise the antimicrobial host immune response, which was also suggested by our own albeit limited mortality data. PTX added to antibiotics might under certain circumstances even support bacterial clearance, and it might protect the host from inflammatory organ injury through the promotion of an anti-inflammatory milieu. On the other hand, adjunctive PTX might exert microbe-specific changes in the host immune response to sepsis. In a model of *Staphylococcus epidermidis*-induced murine neonatal sepsis that was further potentiated by hypoxic-ischemic brain injury, vancomycin reduced bacterial growth and cytokine production in plasma and alleviated the resulting brain injury, whereas the addition of PTX did not provide any additional beneficial effects (58). Likewise, whereas PTX alone or combined with GENT significantly suppressed *E. coli*-induced TNF and IL-1β expression in newborn cord blood monocytes *in vitro*, this agent only led to a minor decrease in *S. epidermidis*-induced IL-1β without modifying intramonocytic TNF expression, and did not alter the expression of any *S. epidermidis*-induced cytokine-encoding genes (18). According to these animal and limited human studies and our own findings, PTX may be a beneficial and feasible adjuvant therapy for neonatal sepsis due to its ability to reduce systemic and peripheral organ inflammation without compromising antibacterial host immunity.

Sex differences in immune function such as distinct vaccine responses, autoimmune diseases, or cancer, is a well-recognized phenomenon and has been extensively reviewed (30, 59, 60). Sex-specific differences in immune functions appear to play a role in the increased manifestation and severity of many complications among male preterm neonates such as perinatal brain injury, bronchopulmonary dysplasia and sepsis (29). To the best of our knowledge, no sex differences in the anti-inflammatory immune responses of adjunctive PTX to infections have been reported to date. Since our study was not powered for the detection of sex differences in cytokine responses, it is possible that small differences in immune responses to our treatment interventions might have remained undetected. Since we did not apply multiple comparison correction to this exploratory analysis of sex-specific cytokine responses, the statistical differences in IL-1β and IL-6 tissue concentrations observed between male and female neonatal mice for several treatment conditions might have occurred by chance alone. The observation that (GENT + PTX) did not suppress IL-6 and IL-1β as much in male *vs*. female neonatal mice in several organ tissues warrants further investigation in future studies.

Our study is novel and has important strengths. We employed a rigorously validated murine neonatal sepsis model, consisting of high quality intravenous live bacterial injections on the first day of life, thereby mimicking human preterm neonatal sepsis (61). Our study demonstrated substantial differences in the effects of adjunctive PTX on systemic *vs*. peripheral organ inflammation as well as organ-specific responses, such that PTX inhibited plasma TNF and enhanced plasma IL-10 concentrations while primarily increasing IL-10 production in organ tissues with little or no effect on tissue TNF levels. The IL-10-promoting effect of PTX might be particularily relevant in the brain, which showed the highest TNF-to-IL-10 concentrations compared to other organ tissues among untreated septic neonatal mice. Our results indicate that the combination of PTX with antibiotics such as GENT might further improve the anti-inflammatory efficacy of these agents. Anti-inflammatory effects of adjunctive PTX to antibiotics were achieved at different time points in relation to sepsis duration, whether anti-inflammatory and antimicrobial treatment was administered simultaneously, early or late after sepsis initiation. And importantly, the shift of bacterial sepsis-induced cytokine responses toward an anti-inflammatory mileu was achieved without an increase of bacterial growth and mortality, suggesting that the adjunctive use of PTX to antibiotics for neonatal sepsis might be safe and beneficial.

On the other hand, our study has several limitations. Our findings were based on murine neonatal *E. coli* sepsis, the most frequently encountered organism of early-onset sepsis in the human neonate. However, the effects of PTX on immune responses in human newborns and those induced by other neonatal pathogens, including the Gram-positive organisms *S. epidermidis* and *S. aureus, Candida* or viral infections such as *Herpes simplex*, will need to be investigated prior to recommending this agent as empiric adjunctive therapy for newborn sepsis syndromes. Neonatal immunity changes rapidly over the course of the first several weeks of life, and preterm neonates are especially vulnerable to microbial infections and infection-induced organ damage including brain injury. Therefore, neonatal sepsis models investigating the effects of adjunctive anti-inflammatory treatment regimens should also study the effects on different gestational and post-natal age groups. As demonstrated in our current study as well as our previous *in vitro* experiments on human cord blood (18), combinations of PTX with antibiotics may lead to different treatment interactions including additive or synergistic anti-inflammatory responses. Our observations pave the way for future studies to: (a) investigate additional antimicrobial agents, such as those with intrinsic anti-inflammatory mechanisms of action (e.g., azithromycin) (62); and (b) assess the potential impact of PTX and other anti-inflammatory agents on metabolites and metabolic pathways relevant to the progression and severity of sepsis, such as lactate. Our study examined the short-term effects of adjunctive PTX on microbe-induced inflammatory cytokine responses and bacterial growth in murine neonatal sepsis. However, longitudinal survival and outcome studies of adjunctive PTX treatment are needed, as well as comprehensive studies on the structural and functional outcome of organs that are adversely affected by neonatal sepsis such as the brain.

In summary, our study demonstrated that adjunctive PTX when added to GENT inhibited *E. coli*-induced TNF and enhanced *E. coli*-induced IL-10 production in blood plasma while also increasing anti-inflammatory IL-10 concentrations in peripheral organ tissues including the brain. This PTX-induced shift toward anti-inflammatory immune responses was achieved whether treatment was administered simultaneously, early or late in relation to sepsis initiation, and without increasing the bacterial burden or mortality in septic neonatal mice. To the extent that sepsis-induced organ damage in newborns is driven by TNF and other pro-inflammatory mediators, our observations raise the possibility that PTX might be a safe and effective anti-inflammatory adjunctive agent to treat sepsis.

## Data Availability Statement

The raw data supporting the conclusions of this article will be made available by the authors, without undue reservation.

## Ethics Statement

The animal study was reviewed and approved by Institutional Animal Care and Use Committee, Stony Brook University, Stony Brook, NY.

## Author Contributions

ES initiated the project, performed formal data analysis, and wrote the original manuscript. ES, ED-N, OL, and BF conceptualized the study and developed the hypothesis and design. ES, ED-N, and LO developed the methodology. LO, ES, ED-N, and MR performed and validated the experiments. OL, BF, and ED-N critically reviewed and edited the manuscript. All authors reviewed the final version of the manuscript.

## Conflict of Interest

OL is a named inventor on patents relating to the anti-infective bactericidal/permeability-increasing protein, human *in vitro* systems that model age-specific immunity, and vaccine adjuvants. The remaining authors declare that the research was conducted in the absence of any commercial or financial relationships that could be construed as a potential conflict of interest.
