# Supplementary material for: A Neonatal Murine Escherichia coli Sepsis Model Demonstrates That Adjunctive Pentoxifylline Enhances the Ratio of Anti- vs. Pro-inflammatory Cytokines in Blood and Organ Tissues
Source: Front Immunol. 2020 Sep 23;11:577878. doi: 10.3389/fimmu.2020.577878 (PMC7538609; doi:10.3389/fimmu.2020.577878)
Supplement: Supplementary file 1 [file Image_1.PDF]

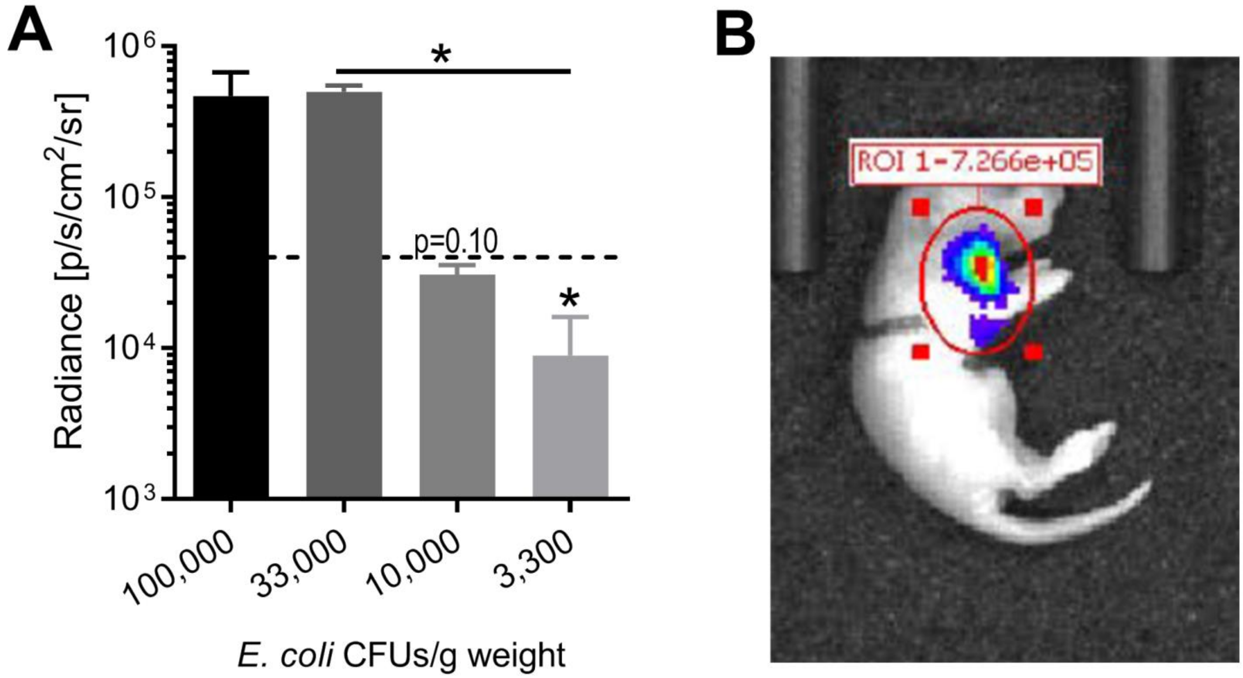

Supplementary Figure 1: Standardization of optical imaging of bioluminescent *E. coli* injections in neonatal mice. Pups were injected with decreasing bacterial loads of bioluminescent *E. coli* subcutaneously into the right neck area, followed by optical imaging. (A) The average radiance measured shortly after injections correlated highly with the injected bacterial inoculum (Spearman correlation $r = +0.91$, $p < 0.001$, $n = 3$ each). Significant differences between median (IQR) radiance of mice injected with 10^5 CFUs/g weight subcutaneously, i.e. the standard bacterial load used for IV injections in this study, compared to decreasing *E. coli* CFUs ($3.3x10^4$, 10^4, and $3.3x10^3$ CFUs, respectively) and between individual bacterial loads were as indicated: $^*p<0.05$, 2-sided Kruskal-Wallis test. The interrupted line depicts the cut-off value used to define successful IV bacterial injections of *E. coli* 10^5 CFUs/g weight in this study. (B) Representative image of a failed IV injection of bioluminescent *E. coli*, demonstrating the ROI placed over the right neck area for radiance measurement.
