# Supplementary material for: A Neonatal Murine Escherichia coli Sepsis Model Demonstrates That Adjunctive Pentoxifylline Enhances the Ratio of Anti- vs. Pro-inflammatory Cytokines in Blood and Organ Tissues
Source: Front Immunol. 2020 Sep 23;11:577878. doi: 10.3389/fimmu.2020.577878 (PMC7538609; doi:10.3389/fimmu.2020.577878)
Supplement: Supplementary file 2 [file Table_1.DOCX]

**Supplementary Table 1: Effect of GENT and/or PTX on *E. coli* CFUs in murine neonatal sepsis analyzed by sex.** Neonatal mice < 24 h old were injected IV with *E. coli* 10^5^ CFU/g body weight, followed by early (1.5 h) IP injection of SAL, GENT, PTX or (GENT+PTX). After an additional 4 h of incubation, i.e. 5.5 h from the time of sepsis initiation, mice were euthanized, and blood and homogenized organ tissues serially plated for bacterial counting. Median (IQR) CFU counts for each organ and treatment condition and separate for both sexes were calculated. 2-sided Mann-Whitney *U* tests were indicated; non-significant (ns).

| **Treatment** | **Organ** | **Sex** | **N** | **CFUs / ml or mg tissue** | | | |
| --- | --- | --- | --- | --- | --- | --- | --- |
|  |  |  |  | **Median** | **IQR** | | **Mann-Whitney *U*** |
| **SAL** | **Blood** | **female** | 14 | 3,200,000 | 1,221,000 - 4,454,500 | ns | |
|  |  | **male** | 10 | 2,175,000 | 262,325 - 2,775,000 |  |  |
|  | **Lung** | **female** | 22 | 7,978 | 1,514 - 19,391 | ns | |
|  |  | **male** | 12 | 3,489 | 1,260 - 35,528 |  |  |
|  | **Liver** | **female** | 22 | 7,651 | 2,084 - 15,023 | ns | |
|  |  | **male** | 12 | 3,133 | 930 - 10,064 |  |  |
|  | **Spleen** | **female** | 22 | 1,960 | 406 - 4,354 | ns | |
|  |  | **male** | 12 | 591 | 258 - 2,426 |  |  |
|  | **Brain** | **female** | 22 | 15 | 4 - 28 | ns | |
|  |  | **male** | 12 | 25 | 7 - 78 |  |  |
| **GENT** | **Blood** | **female** | 12 | 1,500 | 775 - 5,500 | ns | |
|  |  | **male** | 5 | 300 | 150 - 3,300 |  |  |
|  | **Lung** | **female** | 13 | 17 | 8 - 30 | ns | |
|  |  | **male** | 5 | 17 | 0 - 95 |  |  |
|  | **Liver** | **female** | 13 | 100 | 3 - 1,355 | ns | |
|  |  | **male** | 5 | 10 | 0 - 410 |  |  |
|  | **Spleen** | **female** | 13 | 10 | 4 - 98 | ns | |
|  |  | **male** | 5 | 2 | 0 - 31 |  |  |
|  | **Brain** | **female** | 13 | 1 | 0 - 1 | ns | |
|  |  | **male** | 5 | 0 | 0 - 1 |  |  |
| **GENT+PTX** | **Blood** | **female** | 10 | 4,000 | 600 - 6,475 | ns | |
|  |  | **male** | 10 | 1,800 | 600 - 4.600 |  |  |
|  | **Lung** | **female** | 14 | 29 | 5 - 85 | ns | |
|  |  | **male** | 13 | 18 | 2 - 328 |  |  |
|  | **Liver** | **female** | 14 | 61 | 4 - 385 | ns | |
|  |  | **male** | 13 | 106 | 29 – 1,196 |  |  |
|  | **Spleen** | **female** | 14 | 9 | 2 - 21 | ns | |
|  |  | **male** | 13 | 46 | 8 - 193 |  |  |
|  | **Brain** | **female** | 14 | 0 | 0 - 1 | ns | |
|  |  | **male** | 13 | 1 | 0 - 40 |  |  |
| **PTX** | **Blood** | **female** | 10 | 3,840,000 | 2,150,000 - 6,935,000 | ns | |
|  |  | **male** | 10 | 2,300,000 | 450,850 - 5,710 |  |  |
|  | **Lung** | **female** | 12 | 5,326 | 2,074 - 14,575 | ns | |
|  |  | **male** | 11 | 2,480 | 1,287 - 12,458 |  |  |
|  | **Liver** | **female** | 12 | 6,633 | 1,886 - 14,111 | ns | |
|  |  | **male** | 11 | 2,655 | 118 - 4,436 |  |  |
|  | **Spleen** | **female** | 12 | 502 | 256 - 1,346 | ns | |
|  |  | **male** | 11 | 631 | 384 - 2,707 |  |  |
|  | **Brain** | **female** | 12 | 17 | 7 - 57 | ns | |
|  |  | **male** | 11 | 5 | 3 - 34 |  |  |
