# Supplementary material for: A Neonatal Murine Escherichia coli Sepsis Model Demonstrates That Adjunctive Pentoxifylline Enhances the Ratio of Anti- vs. Pro-inflammatory Cytokines in Blood and Organ Tissues
Source: Front Immunol. 2020 Sep 23;11:577878. doi: 10.3389/fimmu.2020.577878 (PMC7538609; doi:10.3389/fimmu.2020.577878)
Supplement: Supplementary file 3 [file Table_2.DOCX]

**Supplementary Table 2: Effects of PTX on *E. coli*-induced cytokines in male and female pups.**

| **Organ** | **Sex** | **N** | **TNF** | | | **IL-1β** | | |
| --- | --- | --- | --- | --- | --- | --- | --- | --- |
|  |  |  | **Mean** | **± SEM** | **Median** | **Mean** | **± SEM** | **Median** |
| **Plasma** | **female** | 12 | 6,735 | ±1,615 | 6,150 | 46 | ±13 | 39 |
|  | **male** | 11 | 6,078 | ±3,102 | 4,447 | 93 | ±40 | 31 |
| **Lung** | **female** | 12 | 533 | ±190 | 367 | 153 | ±13 | 151 |
|  | **male** | 11 | 576 | ±212 | 269 | 137 | ±25 | 121 |
| **Liver** | **female** | 12 | 30,177 | ±8,475 | 21,618 | 453 | ±70 | 398 |
|  | **male** | 11 | 23,993 | ±9,462 | 10,558 | 541 | ±180 | 297 |
| **Spleen** | **female** | 12 | 3,549 | ±844 | 3,577 | 392 | ±91 | 313 |
|  | **male** | 10 | 6,057 | ±3,556 | 1,996 | 360 | ±77 | 321 |
| **Brain** | **female** | 12 | 527 | ±132 | 453 | 16 | ±4 | 10 |
|  | **male** | 11 | 650 | ±148 | 743 | 20 | ±4 | 17 |
|  | | | | | | | | |
| **Organ** | **Sex** | **N** | **IL-6** | | | **IL-10** | | |
|  |  |  | **Mean** | **± SEM** | **Median** | **Mean** | **± SEM** | **Median** |
| **Plasma** | **female** | 12 | 41,766 | ±5,998 | 45,870 | 7,566 | ±1,230 | 7,889 |
|  | **male** | 11 | 40,405 | ±6,051 | 45,042 | 10,168 | ±1,888 | 7,348 |
| **Lung** | **female** | 12 | 1,809 | ±268 | 1,933 | 797 | ±131 | 781 |
|  | **male** | 11 | 2,550 | ±583 | 1,823 | 1,182 | ±282 | 1,160 |
| **Liver** | **female** | 12 | 969 | ±138 | 945 | 1,511 | ±290 | 1,392 |
|  | **male** | 11 | 1,062 | ±184 | 915 | 2,046 | ±543 | 1,623 |
| **Spleen** | **female** | 12 | 896 | ±142 | 899 | 393 | ±64 | 362 |
|  | **male** | 10 | 1,177 | ±373 | 784 | 464 | ±150 | 233 |
| **Brain** | **female** | 12 | 241 | ±103 | 135 | 42 | ±7 | 39 |
|  | **male** | 11 | 564 | ±409 | 106 | 108 | ±49 | 53 |

Neonatal mice < 24 h old were injected IV with *E. coli* 10^5 CFU/g body weight, followed by early (1.5 h) IP injection of PTX. After an additional 4 h of incubation, i.e. 5.5 h from the time of sepsis initiation, mice were euthanized, and plasma and homogenized organ tissue cytokines were measured. Mean (± SEM) and median cytokine concentrations in pg/ml (plasma) or pg/mg protein for each organ and treatment condition and separate for both sexes were calculated. 2-sided Welch *t* tests and Mann-Whitney *U* tests did not show any sex differences in cytokine production in plasma and organs of septic newborn mice treated with PTX alone.
